# Hydroxyl radical-induced formation of highly oxidized organic compounds

**DOI:** 10.1038/ncomms13677

**Published:** 2016-12-02

**Authors:** Torsten Berndt, Stefanie Richters, Tuija Jokinen, Noora Hyttinen, Theo Kurtén, Rasmus V. Otkjær, Henrik G. Kjaergaard, Frank Stratmann, Hartmut Herrmann, Mikko Sipilä, Markku Kulmala, Mikael Ehn

**Affiliations:** 1 Leibniz-Institut für Troposphärenforschung (TROPOS), Permoserstraße 15, 04318 Leipzig, Germany; 2 Department of Physics, University of Helsinki, Helsinki 00014, Finland; 3 Department of Chemistry, University of Helsinki, Helsinki 00014, Finland; 4 Department of Chemistry, University of Copenhagen, Copenhagen 2100, Denmark

## Abstract

Explaining the formation of secondary organic aerosol is an intriguing question in atmospheric sciences because of its importance for Earth's radiation budget and the associated effects on health and ecosystems. A breakthrough was recently achieved in the understanding of secondary organic aerosol formation from ozone reactions of biogenic emissions by the rapid formation of highly oxidized multifunctional organic compounds via autoxidation. However, the important daytime hydroxyl radical reactions have been considered to be less important in this process. Here we report measurements on the reaction of hydroxyl radicals with α- and β-pinene applying improved mass spectrometric methods. Our laboratory results prove that the formation of highly oxidized products from hydroxyl radical reactions proceeds with considerably higher yields than previously reported. Field measurements support these findings. Our results allow for a better description of the diurnal behaviour of the highly oxidized product formation and subsequent secondary organic aerosol formation in the atmosphere.

## Abstract (web summary)

Secondary organic aerosols are important contributors to the Earth's radiation budget, however questions remain about their formation from highly-oxidized precursors. Here the authors show that the daytime reaction of hydroxyl radicals with α- and β-pinene is a greater source of highly-oxidized products than previously assumed.

## Graphical abstract

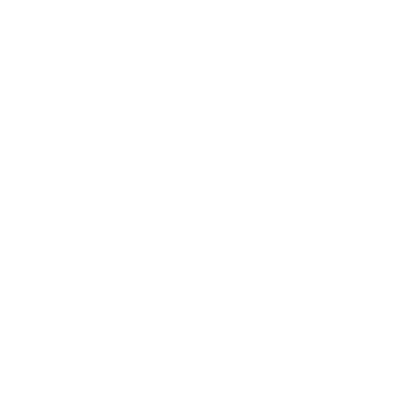

Atmospheric aerosol particles play important roles in the climate^1^,^2^, human health^3^ and ecosystems. It is known that a dominant source of atmospheric aerosol particles is formation via oxidation of inorganic (for example, SO_2_) and organic (for example, α- and β-pinene) precursor gases resulting in low vapour pressure reaction products^4^. However, both fundamental and quantitative knowledge, especially concerning the chemical pathways leading to the formation of aerosol particles from organic precursor gases, are still missing.

Ehn *et al.*^5^ conclusively demonstrated the formation of highly oxidized multifunctional organic compounds (HOMs) from the ozonolysis of α-pinene and their importance to secondary organic aerosol (SOA) formation. HOMs can be partly classified as extremely low-volatility organic compounds^6^ due to the expected low vapour pressures. The remaining fraction can be attributed to low- or semi-volatile organic compounds. An exact determination of the vapour pressure of HOMs is currently impossible preventing a more accurate classification. The HOM detection became feasible by the latest developments of online mass spectrometric techniques^7^,^8^. Other terpene ozonolysis studies^9^,^10^,^11^, using the same detection technique, have confirmed the findings by Ehn *et al.*^5^ and discovered that HOM formation with up to 12 O atoms in the molecules proceeds on a time scale of seconds at atmospheric reactant concentrations. More recently, first indications for the presence of HOMs from α-pinene ozonolysis were found in the particle phase^12^.

The above observations suggest that especially the ozone reaction with terpenes is responsible for rapidly formed low-volatility SOA precursors. Up to now, laboratory studies^5^,^9^,^10^, all using the nitrate ionization technique, indicate a minor importance of OH radical-driven HOM generation compared with ozonolysis. Estimated molar HOM yields of <1% (ref. 5) and 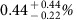
(10) from OH+α-pinene are reported using total signal measurements, that is, all appearing signals in a selected mass-to-charge range were taken into account without a specification of the respective reaction product. Atmospheric measurements, however, point to dominant particle formation and growth during daytime, indicating that there must be a large source of low-volatility organic species that is connected to the photochemistry^4^. The most important daytime oxidant in the atmosphere is the OH radical, whereas nighttime oxidation is dominated by ozone and the NO_3_ radical^13^.

The present work represents a specific study on HOM formation from the OH radical initiated oxidation of the most abundant monoterpenes emitted by vegetation, α- and β-pinene^14^. We measure the ‘early' HOMs, that is, the highly oxidized RO_2_ radicals, which represent the intermediates finally forming closed-shell HOMs in the atmosphere via different reaction pathways. These reaction pathways are, for example, bimolecular reactions with NO, NO_2_, HO_2_ and other RO_2_ radicals or unimolecular reaction steps^13^. We find that these RO_2_ radicals can be detected with good sensitivity^9^,^15^, enhancing our ability to understand the HOM formation process in more detail. In our experiments designed to probe the highly oxidized RO_2_ radical generation, consecutive bimolecular RO_2_ radical reactions are unimportant because of low concentrations (<10^7^ molecules cm^−3^) and short reaction times in the range of 3.0–7.9 s. The HOM detection is carried out by means of chemical ionization–atmospheric pressure interface–time-of-flight (CI-APi-TOF) mass spectrometry (Airmodus, Tofwerk) with a detection limit of ∼10^4^ molecules cm^−3^. A recent study indicated that nitrate ionization, the technique used in almost all HOM studies so far, may not be sensitive to all key HOM compounds^15^. Therefore, the experimental approach applied here comprises a set of four different reagent ions for product identification, that is, nitrate (NO_3_^−^), lactate (CH_3_CH(OH)COO^−^), pyruvate (CH_3_C(O)COO^−^) and acetate (CH_3_COO^−^), to probe the ion-specific detection efficiency. The investigations are conducted in a free-jet flow system at 295±2 K and at atmospheric pressure^15^. Additional experiments with elevated HO_2_ and RO_2_ radical concentrations or NO additions are conducted, to probe qualitatively the closed-shell HOM formation starting from the highly oxidized RO_2_ radicals. In conclusion, our results show the importance of the OH radical-induced formation of HOMs from monoterpenes. This finding allows a better description of the diurnal cycle of SOA formation by available HOMs formed either from the ozonolysis or via OH radical reactions.

## Results

### Reagent ion-dependent detection efficiency

**Figure 1:**
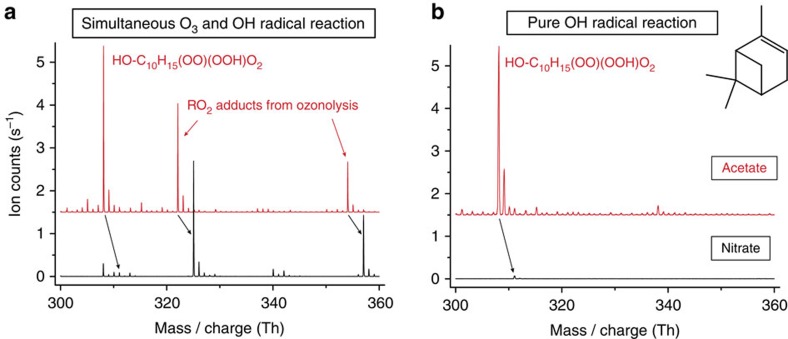
Recorded mass spectra using acetate or nitrate for ionization. The detected RO_2_ radicals from the oxidation of α-pinene appear as adducts with the reagent ions. Signals of nitrate adducts (black) are shifted by three mass units compared to the corresponding acetate adduct signals (red). The spectra obtained with acetate ionization are offset by 1.5 s^−1^ for more clarity. The reaction time in all experiments was 7.9 s. (**a**) Spectra obtained from α-pinene ozonolysis for identical conditions [O_3_]=6.1 × 10^11^ and [α-pinene]=1.0 × 10^12^ molecules cm^−3^. Simultaneous O_3_ and OH radical reaction takes place due to OH radical production from ozonolysis. (**b**) Spectra from the pure OH+α-pinene reaction using H_2_O_2_ photolysis for OH radical formation, [H_2_O_2_]∼1 × 10^14^ and [α-pinene]=5.0 × 10^12^ molecules cm^−3^. The signal at nominal 309 Th (first isotope signal of the RO_2_ radical) can be partly influenced by the corresponding hydroperoxide.

In the experiments, a strong enhancement of the signals attributed to the OH radical-derived highly oxidized RO_2_ radicals occurred after switching the reagent ion from nitrate to lactate, pyruvate or acetate. All detected RO_2_ radicals were exclusively formed in the flow system as shown by experiments for characterizing the measurement system, (Supplementary Figs 1–3 and Supplementary Note 1). The spectra in Fig. 1a,b show the comparison of results from the ozonolysis of α-pinene (simultaneous O_3_ and OH radical reaction) and from the pure OH+α-pinene reaction, respectively, using either nitrate or acetate for ionization. The occurrence of the strong signal at nominal 308 Th using acetate ionization (at nominal 311 Th for nitrate ionization) in both sets of experiments demonstrates that this product is formed from the OH radical attack on α-pinene without a contribution of any ozone reactions. In contrast to the OH radical-derived RO_2_ radicals, the signal strength of RO_2_ radicals from the simultaneous O_3_+α-pinene reaction remained almost unchanged switching from nitrate to acetate (Fig. 1a). A strong signal at nominal 308 Th appeared also in the case of the OH+β-pinene reaction using acetate ionization, in line with the findings from the α-pinene system (Supplementary Fig. 4).

**Figure 2:**
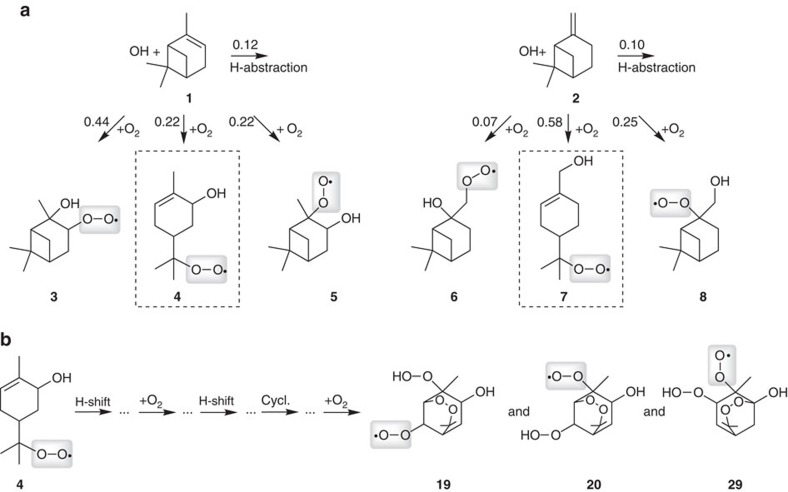
Proposed reaction scheme for the formation of highly oxidized RO_2_ radicals. (**a**) Formation of first RO_2_ radicals from atmospheric OH radical reaction of α- and β-pinene. (**b**) Possible structures of highly oxidized RO_2_ radicals **19**, **20** and **29**, HO-C_10_H_15_(OO)(OOH)O_2_, formed from RO_2_ radical **4** via a reaction sequence of isomerization steps (H-shift and cyclization) followed by O_2_ addition. Branching ratios stated in **a** were taken from refs 19.

The predominant signal of the OH radical-derived RO_2_ radicals, observed by means of all four reagent ions, is consistent with the elemental formula HO-C_10_H_16_O_6_. Experiments in the presence of heavy water allow the determination of acidic H atoms in the molecules (equal to the number of OH and OOH groups) by H/D exchange measuring the resulting signal shift in the mass spectrum^16^, (Supplementary Fig. 5). The analysis revealed two acidic H atoms in HO-C_10_H_16_O_6_, indicating a chemical composition of **H**O-C_10_H_15_(OO)(OO**H**)O_2_. Another composition of this species, including other functional groups such as ether or carboxylic groups, is implausible and would be contrary to the current knowledge of possible elementary steps in these reaction systems^5^,^9^,^11^,^15^,^16^. It should be noted that there is one acidic H atom less than expected, if all inserted O_2_ into the molecule (beside the peroxy O_2_ of the RO_2_ radical) were present as OOH groups^9^,^15^,^16^. It can be speculated that the additional ‘(OO)' group stands for an endo-peroxide generated via ring closure of an unsaturated RO_2_ radical. Such a process was predicted by theoretical calculations^17^ and qualitatively confirmed by a product study from a chamber experiment^18^. The initial, unsaturated RO_2_ radicals, species **4** and **7**, are formed from the OH radical reaction of α- and β-pinene with high yields (Fig. 2)^19^,^20^. Possible reaction pathways for the generation of the highly oxidized RO_2_ radicals from the OH+α-pinene reaction have been proposed (Supplementary Figs 6–9). The pathways include an initial intramolecular H-shift^5^,^9^,^15^,^16^,^21^, followed by O_2_ addition leading to RO_2_ radicals with five O atoms. Then a very rapid H-shift involving the OOH group^22^ takes place followed by an endo-cyclization and a next O_2_ addition to get the RO_2_ radicals **19**, **20** and **29** containing seven O atoms each. Based on quantum chemical calculations (Supplementary Tables 1–3 and Supplementary Note 2), the RO_2_ radicals **19**, **20** and **29**, all with the chemical composition HO-C_10_H_15_(OO)(OOH)O_2_, can be formed on a seconds time scale starting from the RO_2_ radical **4** as illustrated in a simplified way in Fig. 2b. The calculations furthermore indicate that **19**, **20** and **29** are relatively stable with respect to further isomerization steps (H-shifts). For β-pinene, similar reaction steps are assumed. An additional minor signal appeared in the β-pinene spectra that was attributed to HO-C_10_H_16_O_8_ (Supplementary Fig. 4). A composition **H**O-C_10_H_14_(OO)(OO**H**)_2_O_2_ can be assumed based on the H/D exchange results, which indicated three acidic H atoms in the molecule. It should be noted that the reaction sequence of RO_2_ radical isomerization (H-shift or cyclization) followed by O_2_ addition (Fig. 2) represents a reaction principle well known in low-temperature combustion chemistry^23^.

### Estimated molar HOM yields

**Figure 3:**
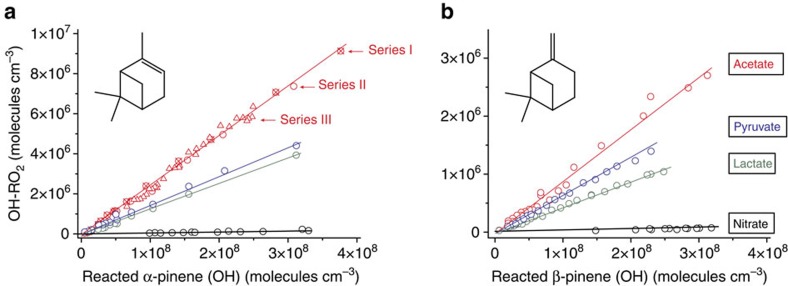
Estimated RO_2_ radical concentrations as a function of reacted α- and β-pinene. The given concentrations for the different reagent ions were obtained using a calibration factor from absolute sulphuric acid calibration that is in line with the calculated, lower end calibration factor of an ion-molecule reaction. Reagent ions (colour coding): nitrate (black), lactate (green), pyruvate (blue) and acetate (red). The reaction time in all experiments was 7.9 s. (**a**) α-Pinene reaction: series I and II from acetate ionization and the results from the other reagent ions were obtained from α-pinene ozonolysis with OH radical formation via O_3_+α-pinene. [O_3_]=6.1 × 10^11^ and [α-pinene]=(1.2–81) × 10^10^ molecules cm^−3^. Series III (acetate ionization) shows findings from combined TME/α-pinene ozonolysis. O_3_+TME generates additional OH radicals. [O_3_]=9.1 × 10^11^, [TME]=(1.8–110) × 10^9^, [α-pinene]=1.0 × 10^11^ molecules cm^−3^. (**b**) β-Pinene reaction: combined TME/β-pinene ozonolysis with preferred OH radical formation via the O_3_+TME reaction, [O_3_]=9.1 × 10^11^, [TME]=(1.8–95) × 10^9^, [β-pinene]=1.05 × 10^11^ molecules cm^−3^.

The estimated RO_2_ radical concentrations for both terpenes as a function of reacted α- and β-pinene gave a linear response for terpene conversions smaller than 4 × 10^8^ molecules cm^−3^ (Fig. 3). This behaviour confirmed the absence of significant bimolecular RO_2_ radical reactions for these experimental conditions. Different measurement series (I–III) revealed consistent and reproducible results. The stated radical concentrations, and consequently the yields, represent estimated lower end values for the different ionization schemes. Resulting molar yields of highly oxidized RO_2_ radicals from OH+α-pinene are 2.4±0.1% (acetate ionization) and 0.052±0.006% (nitrate ionization). The corresponding values for OH+β-pinene are 0.90±0.03% and 0.022±0.001%, respectively. Given error limits comprise statistical errors only.

The estimated RO_2_ radical yields deduced from nitrate ionization are about a factor of 40 smaller than those from acetate ionization pointing to more stable (OH-RO_2_)·acetate adducts compared with the corresponding nitrate adducts. (Note: the factor of 40 is substance specific and not generally valid.) Theoretical calculations on the cluster stability of model RO_2_ radicals with nitrate or acetate have been performed, which support the experimental findings of this study (Supplementary Figs 10 and 11, Supplementary Table 4 and Supplementary Note 3). When comparing the experiments using ionization by lactate and pyruvate with those applying acetate, the results differ by a factor smaller than two, indicating similar cluster stabilities for the organic reagent ions. In the case of the ozonolysis-derived RO_2_ radicals, the four reagent ions yielded almost the same sensitivities within a factor of 1.6 (Supplementary Fig. 12).

### Additional runs with NO and increased RO_2_ concentrations

Furthermore, we tested the reactivity of the detected RO_2_ radicals from the OH+α-pinene reaction towards NO and measured the resulting product formation for atmospherically relevant NO concentrations of (5.6–280) × 10^8^ molecules cm^−3^ (Supplementary Fig. 13). An increasing signal, HO-C_10_H_15_(OO)(OOH)ONO_2_, appeared with rising NO concentrations according to the organic nitrate formation via RO_2_+NO→RONO_2_ starting from HO-C_10_H_15_(OO)(OOH)O_2_ (refs 9, 15, 24). Other signals were tentatively attributed to reaction products of the corresponding alkoxy radical, HO-C_10_H_15_(OO)(OOH)O, formed in a parallel way via RO_2_+NO→RO+NO_2_. The total amount of products indicates that the HO-C_10_H_15_(OO)(OOH)O_2_ radical formation was not disturbed by the NO additions (Supplementary Fig. 13 and Supplementary Note 4). Thus, the RO_2_ isomerization steps leading to HO-C_10_H_15_(OO)(OOH)O_2_ must be faster than the corresponding RO_2_ reactions with NO, which proceed with pseudo first-order rate coefficients of up to 0.28 s^−1^. This estimate assumes a rate coefficient *k*(NO+RO_2_) of 1 × 10^−11^ cm^3^ per molecule per second^24^. Consequently, the rate coefficients of the RO_2_ isomerization steps forming the RO_2_ radicals **19**, **20** and **29** must be larger than 0.28 s^−1^ in line with the results of theoretical calculations.

H_2_O_2_ photolysis experiments with increased reactant concentrations were conducted in the TROPOS flow tube^25^. For OH+α-pinene, the product formation from the reaction of the highly oxidized RO_2_ radicals with HO_2_ and other RO_2_ radicals were studied (Supplementary Fig. 14a,b). The analysis of the mass spectra recorded for different RO_2_/HO_2_ ratios allowed a qualitative description of the respective reaction products. The reaction of HO-C_10_H_15_(OO)(OOH)O_2_ with HO_2_ yielded the corresponding hydroperoxide according to RO_2_+HO_2_→ROOH+O_2_. The main product from the reaction with other RO_2_ radicals was a species with −15 nominal mass units compared with the precursor RO_2_ radical, probably HO-C_10_H_15_(OO)(OOH)OH. In the mass spectra also signals of C_20_ accretion products^5^,^9^,^11^,^15^,^16^ appeared, which are consistent with the chemical formulas C_20_H_34_O_8_, C_20_H_34_O_10_ and C_20_H_34_O_12_ (Supplementary Fig. 14b). Their formation can be mechanistically explained via RO_2_+R′O_2_→ROOR′+ O_2_. RO_2_ and R′O_2_ represent the peroxy radicals from the OH+α-pinene reaction, which contain either three (species **3**, **4** or **5**), five (species **12**, **13** or **14**) or seven O atoms (species **19**, **20** or **29**).

We calculated the vapour pressure of three closed-shell C_10_ HOMs formed from the reaction of HO-C_10_H_15_(OO)(OOH)O_2_ radicals with HO_2_, NO or other RO_2_ radicals using the increment method SIMPOL.1 (ref. 26) and the COSMO-RS approach^27^. All the estimated vapour pressures of the C_10_ products were below 10^−9^ atm at 295 K. Vapour pressures below 10^−15^ atm were determined for the C_20_ accretion products applying solely the SIMPOL.1 method^26^ (Supplementary Table 5 and Supplementary Note 5). Thus, these highly oxidized products have a low volatility (and are water soluble) and can effectively condense on surfaces. Recently, SOA yields of 17–26% from low-NO α-pinene photooxidation experiments in the Caltech chamber were reported being independent of OH exposure^28^. This finding was not in line with SOA modelling results for this reaction system, indicating the presence of other SOA formation routes, such as an autoxidation mechanism^5^,^9^,^21^, not implemented in modelling schemes yet^28^. Our lower limit molar HOM yield from OH+α-pinene of 2.4% corresponds to a lower limit mass yield of 4.4% (average HOM mass: 250 g mol^−1^). This mass yield can explain at least a fraction of the measured SOA yield from the Caltech experiments^28^ and also from other SOA studies^29^.

### Comparison of laboratory and field measurements

**Figure 4:**
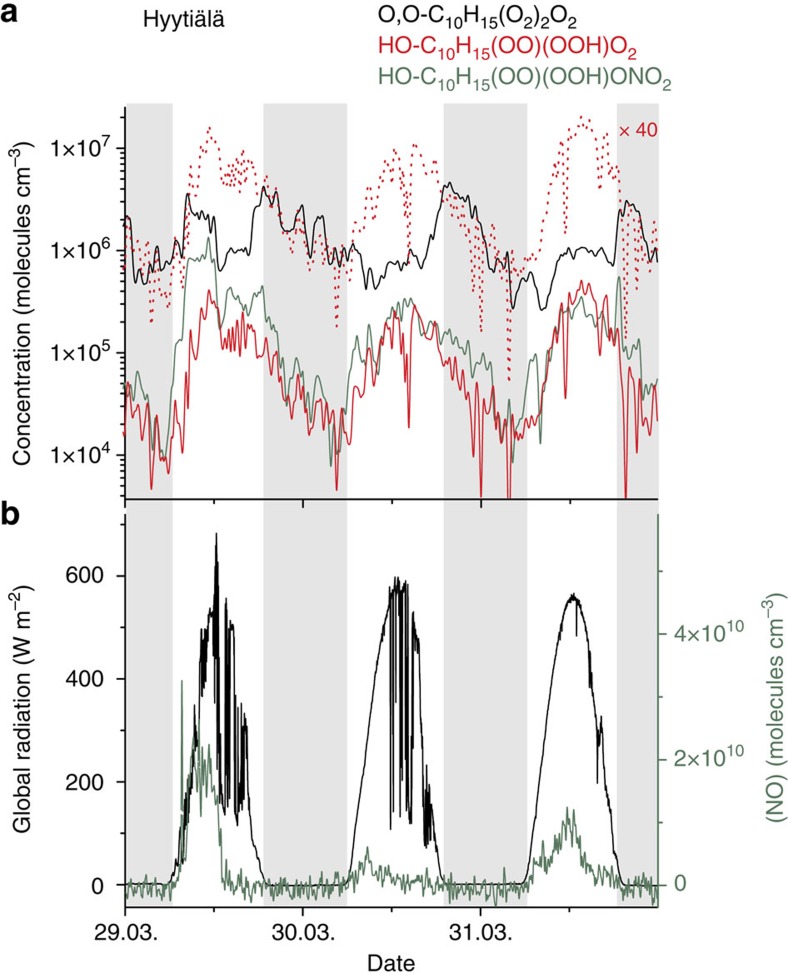
Field measurements at the SMEAR II station. Hyytiälä, Finland, 29–31 March 2011. (**a**) Time series of the estimated concentrations of O,O-C_10_H_15_(O_2_)_2_O_2_ radicals (in black) from ozonolysis of terpenes and HO-C_10_H_15_(OO)(OOH)O_2_ radicals (in red) from the OH radical reaction as well as the corresponding organic nitrate (in olive). Full lines show the measurements by the nitrate-CI-APi-TOF mass spectrometer, the dashed red line stands for the corrected HO-C_10_H_15_(OO)(OOH)O_2_ radical trace assuming an enhanced sensitivity by a factor of 40 based on the comparison of results using nitrate or acetate ionization in the laboratory experiments. (**b**) Global radiation is taken as a proxy for OH radicals. Enhanced NO concentration especially in morning hours of 29 March yielded enhanced organic nitrate concentrations.

Furthermore, field measurements from two different sites support the findings from the laboratory studies presented here. Figure 4a shows the time series of the most abundant, highly oxidized RO_2_ radicals from terpene ozonolysis^9^ (in black) and from the OH radical reaction (in red), as well as the corresponding organic nitrate (in green). The corresponding global radiation, serving as a proxy for the OH radical concentration, and the measured NO concentrations are depicted in Fig. 4b. The ozone concentration showed relatively small variation during the whole campaign (Supplementary Fig. 15). The measurements have been done at the boreal research station SMEAR II^30^ in the spring 2011 using nitrate-CI-APi-TOF mass spectrometry. Considering an underestimation of the RO_2_ concentrations from the OH radical reaction by a factor of 40 using nitrate ionization, as experimentally shown in this study, the corrected HO-C_10_H_15_(OO)(OOH)O_2_ radical concentrations (dashed red line) reach peak concentrations of about 10^7^ molecules cm^−3^ at daytime. This level clearly exceeds the ozonolysis-derived RO_2_ radical concentrations. Further support for the atmospheric relevance of the OH radical initiated HOM formation comes from a 10-day measurement campaign at the TROPOS research station in Melpitz^31^ in the summer 2013. Here, the diurnal pattern of the HO-C_10_H_15_(OO)(OOH)O_2_ radical followed the diurnal trend of the global radiation, which was again used as a proxy for OH (Supplementary Fig. 16). This behaviour is consistent with the findings from the SMEAR II station.

Atmospheric lifetimes of RO_2_ radicals regarding the reaction with other RO_2_, HO_2_ and NO are in the range of 1.7–170 min for the individual reactions assuming trace gas concentrations for non-urban conditions of 10^9^ molecules cm^−3^ for RO_2_ and HO_2_ radicals, and NO each, *k*(RO_2_+RO_2_)∼1 × 10^−12^–10^−13^, *k*(HO_2_+RO_2_)∼1 × 10^−11^ and *k*(NO+RO_2_)∼1 × 10^−11^ cm^3^ per molecule per second^24^. Taking all these bimolecular steps together, a RO_2_ lifetime of a few minutes has to be taken into account. The nocturnal HO-C_10_H_15_(OO)(OOH)O_2_ radical decline as given in Fig. 4a, however, indicates a RO_2_ lifetime of a few hours. It could be speculated that (i) changing air masses with changing trace gas composition or (ii) boundary layer effects, or (iii) the non-zero nighttime OH radical reaction with terpenes influenced the nocturnal RO_2_ concentration. In addition, instrumental uncertainties cannot be totally ruled out for the nighttime measurements close to the detection limit.

## Discussion

Our results clearly demonstrate the importance of the OH radical initiated oxidation of monoterpenes for a rapid formation of HOMs in the atmosphere. The findings of this work, together with the known process based on the ozonolysis of biogenic emissions^5^,^8^,^9^, allow a more precise description of the diurnal behaviour of HOM generation and subsequent SOA formation, from the different oxidants. The participation of OH radical reactions for HOM formation is in agreement with the fact that both photochemistry and HOMs seem to be important in atmospheric particle formation processes^5^.

Furthermore, several studies indicate a strong quantitative disagreement between experimentally observed SOA formation from field campaigns and model simulations based on laboratory data^32^,^33^,^34^,^35^. For instance, Russell *et al.*^34^ observed for photooxidation conditions, most likely to be dominated by the OH radical reactions of α- and β-pinene, up to two orders of magnitude higher SOA generation compared with model predictions. The results of our study are able to overcome this discrepancy, at least qualitatively. However, also for urban areas with prevalent anthropogenic emissions, much larger amounts of SOA are reported than predicted by models^35^. Volkamer *et al.*^35^ determined that a significant fraction of the excess SOA was formed from first-generation oxidation products of the anthropogenic emissions. It could be speculated that similar reaction pathways of HOM formation, as shown in our study for OH radical reaction with α- and β-pinene, take place in the course of the degradation of anthropogenic emissions, such as aromatics. However, a simple transfer of our results to other reaction systems is impossible due to the substance-specific reactivity of the RO_2_ isomerization steps. Especially in this field, much more experimental and theoretical works are needed, to clarify the formation pathways of SOA precursors generated from anthropogenic emissions.

## Methods

### Free-jet flow system

The experiments have been performed in a free-jet flow system at a pressure of 1 bar purified air (or O_2_/N_2_ mixtures) and a temperature of 295±2 K (15,36). The reaction time was in the range of 3.0–7.9 s. This set-up allows the investigation of oxidation reaction for atmospheric conditions in absence of wall effects.

The free-jet flow system consists of an outer tube (length: 200 cm, inner diameter: 15 cm) and a moveable inner tube (outer diameter: 9.5 mm) equipped with a nozzle. Ozone or H_2_O_2_ premixed with the carrier gas (5 l min^−1^ STP, standard temperature and pressure) is injected through the inner tube into the main gas stream (95 l min^−1^ STP), which contains the second reactant (α- or β-pinene). Large differences of the gas velocities at the nozzle outflow (nozzle: 15.9 m s^−1^; main flow: 0.13 m s^−1^) and the nozzle geometry ensure rapid reactant mixing downstream the nozzle. Diffusion processes at 1 bar air are too slow to transport a significant fraction of the reaction products out of the centre flow towards the walls within the time range of this experiment (3.0–7.9 s).

Ozone was produced by passing 1–2 l min^−1^ (STP) air through an ozone generator (UVP OG-2) and blended with carrier gas to a total flow of 5 l min^−1^ (STP) taken as the feed for the inner tube. In the case of H_2_O_2_ photolysis experiments, a flow of 0.03–1.0 l min^−1^ air (STP) over a H_2_O_2_ sample (saturator maintained at 273 K) supplied the oxidant feed. Photolysis was carried out downstream the mixing point of the gas streams by means of 8 low-pressure ultraviolet lamps emitting in the range 300–320 nm (Cosmedico Licht GmbH, ARIMED B6).

Additional photolysis experiments were conducted in the TROPOS flow tube^25^ at a temperature of 293±0.5 K using synthetic air as the carrier gas. The first flow-tube section (56 cm) contains the inlet system for the reactant gases (air mixed with α-pinene and H_2_O_2_ taken from a saturator as described before). A second section (344 cm) surrounded by 8 ultraviolet lamps (Hg-lamps made of quartz-glass PN235 with a cutoff wavelength of 210 nm) represents the photolysis zone. The sampling outlets are attached at the non-irradiated end section (∼20 cm). The total gas flow was set at 20 l min^−1^ (STP) resulting in a reaction time of 48 s.

Ozone was followed by means of a gas monitor (Thermo Environmental Instruments 49C). The concentrations of α- and β-pinene and tetramethylethylene (TME) were detected with the help of a proton transfer reaction mass spectrometer (Ionicon, PTR-MS 500)^37^.

All gas flows were set by means of calibrated gas flow controllers (MKS 1259/1179). The chemicals and gases had the following purity: α-pinene (99.5%, Fluka), β-pinene (99.0%, Fluka), TME (2,3-dimethyl-2-butene, 99%, Aldrich), acetic acid (99.5%, Aldrich), lactic acid (∼90%, Merck), pyruvic acid (98%, Fluka), NO (98.5%, Aldrich), N_2_ (99.9997%, AirProducts) and O_2_ (99.9992%, AirProducts). Air was taken from a commercial PSA (Pressure Swing Adsorption) unit with further purification by activated charcoal, 4 Å molecular sieve and subsequently by GateKeeper CE-2500KH084R, Entegris.

### CI-APi-TOF mass spectrometer and HOM quantification

Detection of highly oxidized products was carried out using a CI-APi-TOF mass spectrometer (Airmodus, Tofwerk) sampling the centre flow through a sampling inlet (length: 28 cm, inner diameter: 1.6 cm) with a rate of 10 l min^−1^ (STP). Another sampling inlet with a similar geometry allows dilution of the sample flow by a factor of 7 using arbitrary dilution gases. Applied reagent ions were nitrate (NO_3_^−^), acetate (CH_3_COO^−^), lactate (CH_3_CH(OH)COO^−^) and pyruvate (CH_3_C(O)COO^−^). A flow of 0.5–5 ml min^−1^ air over a concentrated acid sample (HX: nitric acid, acetic acid, lactic acid or pyruvic acid) was added to a 35 l min^−1^ (STP) flow of purified air producing the HX containing sheath air that forms the charger ions, X^−^, (HX)X^−^ and (HX)_2_X^−^, after ionization with a ^241^Am source. Highly oxidized organic products are able to form stable (HOM)X^−^ clusters as already shown for nitrate adducts (X^−^≡NO_3_^−^)^5^,^9^,^11^,^15^,^16^,^38^ and acetate adducts (X^−^≡CH_3_COO^−^)^15^. Proton transfer from the HOM to the charger ion, as described for measurements by acetate ionization^39^,^40^, was found to be negligible in this reaction system. HOM concentrations were determined according to equation (1). The values given in the brackets are the measured ion signals.


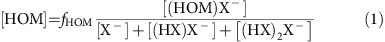


Absolute signal calibration is impossible due to the lack of HOM reference substances. The lower end value of the calibration factor *f*_HOM_ can be calculated considering the (HOM)X^−^ adduct formation in the CI-inlet via reaction (2), *f*_HOM,calc_=1/(*k* × *t* × *f*_inlet_)^5^,^41^, where *k* is the rate coefficient of the ion-molecule reaction, *t* the reaction time and the term *f*_inlet_ considers the sample (HOM) loss in the sampling tube.


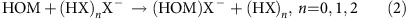


The rate coefficient *k*=*k*_2_ is set to *k*_2_=(2–3) × 10^−9^ cm^3^ per molecule per second, typical for a series of ion-molecule reactions close to the collision limit^42^,^43^. Taking into account a 12% diffusion loss of the sample (HOM) in the short sampling tube (diffusion controlled wall loss for an assumed diffusion coefficient *D*=0.08 cm^2^ s^−1^), *f*_inlet_=0.88 and a reaction time of the ion-molecule reaction *t*=0.2–0.3 s, *f*_HOM,calc_=(1.3–2.8) × 10^9^ molecules cm^−3^ follows. The only reliable absolute calibration at the moment in our system is that used for sulphuric acid detection via H_2_SO_4_+(HNO_3_)_*n*_NO_3_^−^, *n*=0, 1, 2, 3 (refs 44, 45) with a calibration factor *f*_H2SO4,exp_=1.85 × 10^9^ molecules cm^−3^ (ref. 46). This value is in good agreement with the range of *f*_HOM,calc_, the lower end value of *f*_HOM_. By practical reasons (using a definite value of the calibration factor and not a range), *f*_HOM_ in equation (1) was set equal to *f*_H2SO4,exp_. The total uncertainty of the lower end determination of HOM concentration according to equation (1) is estimated with a factor of 2 including changing ion transmission in the considered mass range^7^ as well.

Identical voltage settings in the mass spectrometer were applied for the four ionization techniques (reagent ion: nitrate, acetate, lactate and pyruvate) as optimized for low fragmentation measurements in the nitrate ionization mode.

### Determination of reacted α- and β-pinene

The reaction conditions chosen in the free-jet experiments (low reactant concentrations, reactant conversion: <<1%) did not allow measuring the amount of converted α- and β-pinene. Concentrations of reacted α- and β-pinene from ozonolysis reactions were calculated based on a simple reaction scheme.


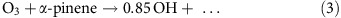



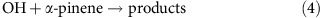



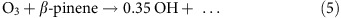



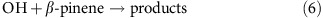



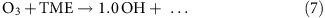



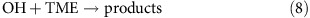


OH radical yields from ozonolysis and the rate coefficients at 295 K were taken from the literature^13^: (unit: cm^3^ per molecule per second) *k*_3_=1.1 × 10^−16^, *k*_4_=5.3 × 10^−11^, *k*_5_=2.24 × 10^−17^, *k*_6_=7.8 × 10^−11^, *k*_7_=1.0 × 10^−15^ and *k*_8_=1.1 × 10^−10^. Other OH radical reactions than those with the fed alkenes are negligible.

Converted α- and β-pinene via the OH radical reaction or via ozonolysis was calculated by numerical integration of the resulting ODE system according to pathways (3)–(8).

Initial reactant concentration of the ozonolysis experiments were:

- pure ozonolysis of α-pinene: [O_3_]=6.1 × 10^11^, [α-pinene]=(1.2–250) × 10^10^ molecules cm^−3^.
- simultaneous ozonolysis of TME/α-pinene: [O_3_]=9.1 × 10^11^, [TME]=(1.8–110) × 10^9^, [α-pinene]=1.0 × 10^11^ molecules cm^−3^.
- simultaneous ozonolysis of TME/β-pinene: [O_3_]=9.1 × 10^11^, [TME]=(1.8–100) × 10^9^, [β-pinene]=1.05 × 10^11^ molecules cm^−3^

The calculation of converted α- and β-pinene from the H_2_O_2_ photolysis experiments was impossible due to the lack of a precise measurement technique for H_2_O_2_. Thus, only qualitative information can be obtained from the photolysis runs.

### Unwanted bimolecular RO_2_ reactions in the free-jet experiment

The basic idea of the ozonolysis-based OH radical experiments performed in the free-jet flow system was to conduct the reaction under conditions of negligible bimolecular RO_2_ reactions. We are not able to measure total RO_2_ and HO_2_ radical concentrations in our experiment. As a conservative estimate, it is assumed that the total RO_2_ as well as the HO_2_ radical concentration did not exceed the amount of reacted terpene of <8 × 10^8^ molecules cm^−3^. Rate coefficient of the RO_2_+RO_2_ and the HO_2_+RO_2_ reactions are assumed to be in the range of *k*(RO_2_+RO_2_)∼1 × 10^−12^–10^−13^ cm^3^ per molecule per second and *k*(HO_2_+RO_2_) ∼ 1 × 10^−11^ cm^3^ per molecule per second^24^. From this, first-order rate coefficients for the stated upper reactant concentration limit are derived as *k*^1st^(RO_2_)<8 × 10^−4^ s^−1^ and *k*^1st^(HO_2_)<8 × 10^−3^ s^−1^. The corresponding lifetimes are >21 min and >2.1 min, respectively. In the case of NO, a background concentration smaller than 1 × 10^8^ molecules cm^−3^ can be deduced from the experiments with NO additions. In those, even for an NO addition of 5.6 × 10^8^ molecules cm^−3^, clear product signals from NO+RO_2_ were visible (Supplementary Fig. 13), which were absent without NO additions. An assumed rate coefficient *k*(NO+RO_2_) of 1 × 10^−11^ cm^3^ per molecule per second^24^ yields a first-order rate coefficients of <1 × 10^−3^ s^−1^ for NO concentrations smaller than 1 × 10^8^ molecules cm^−3^ corresponding to a RO_2_-lifetime with respect to the NO reaction of more than 16 min.

Consequently, any bimolecular reactions of RO_2_ radicals with other RO_2_ radicals, HO_2_ or NO cannot be competitive with the RO_2_ isomerization steps proceeding at a time scale of seconds or less.

### Data availability

The data that support the findings of this study are available from the corresponding author upon reasonable request.

## Additional information

**How to cite this article:** Berndt, T. *et al.* Hydroxyl radical-induced formation of highly oxidized organic compounds. *Nat. Commun.* **7,** 13677 doi: 10.1038/ncomms13677 (2016).

## Supplementary Material

Supplementary Information Supplementary Figures 1-16, Supplementary Tables 1-5, Supplementary Notes 1-5 and Supplementary References

Peer Review File
