# Supplementary material for: Hydroxyl radical-induced formation of highly oxidized organic compounds
Source: Nat Commun. 2016 Dec 2;7:13677. doi: 10.1038/ncomms13677 (PMC5146283; doi:10.1038/ncomms13677)
Supplement: Supplementary Information — Supplementary Figures 1-16, Supplementary Tables 1-5, Supplementary Notes 1-5 and Supplementary References [file ncomms13677-s1.pdf]

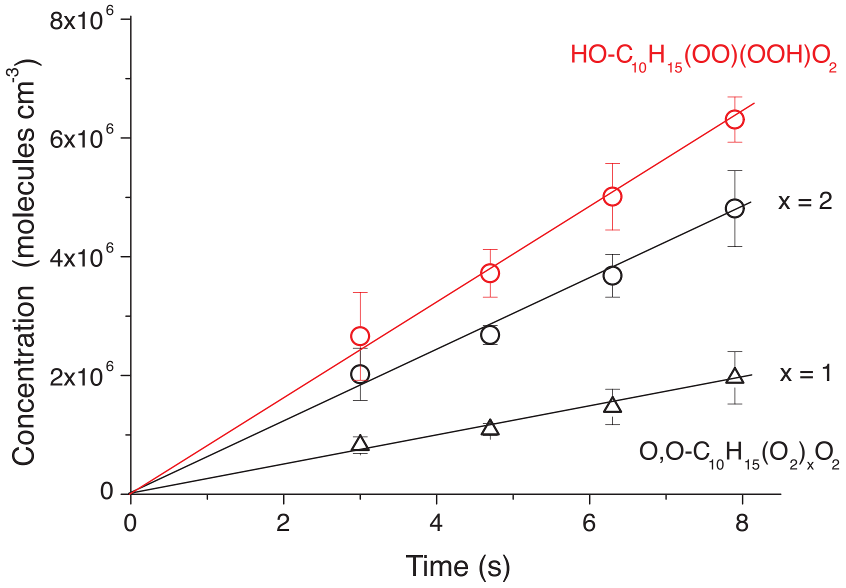

Supplementary Figure 1. Time-dependent RO_2 radical concentrations. Time-dependent RO_2 radical measurements from ozonolysis of α-pinene, $[O_3] = 6.1 \times 10^{11}$, [α-pinene] $= 1.0 \times 10^{12}$ molecules cm^{-3}, using acetate ionization. O,O-$C_{10}H_{15}(O_2)_xO_2$ stands for the RO_2 radical formed from ozonolysis with x inserted O_2 in the molecule (other than the peroxy O_2 of the RO_2 radical) without further specification. The error bars represent two standard deviations of the statistical error from five experiments each.

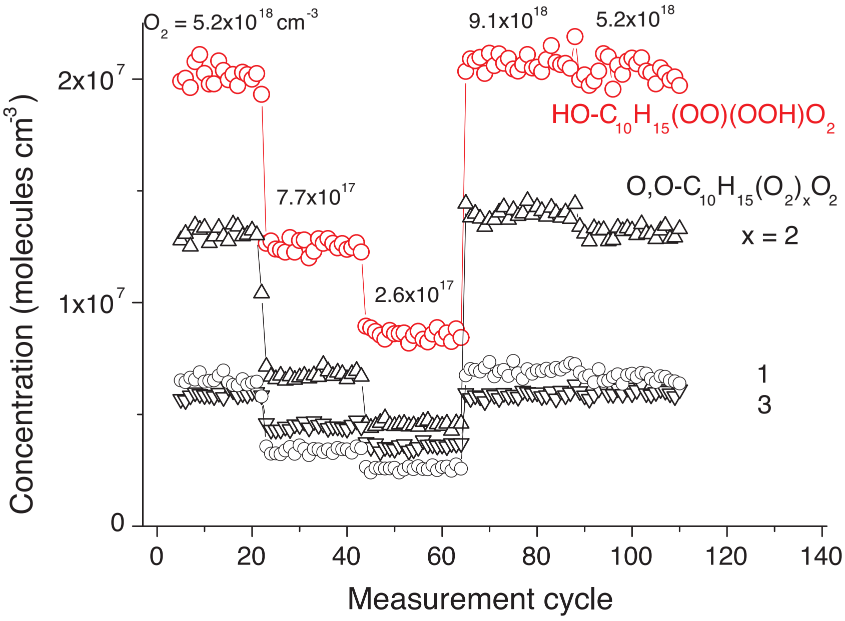

Supplementary Figure 2. O_2 variation in the flow system. O_2-dependent RO_2 radical measurements from ozonolysis of α-pinene, $[O_3] = 6.1 \times 10^{11}$, [α-pinene] $= 2.5 \times 10^{12}$ molecules cm^{-3}, reaction time: 7.9 s, using acetate ionization. Stated O_2 values are the O_2 concentrations in the flow system and in the ionization region. O,O-$C_{10}H_{15}(O_2)_xO_2$ stands for the RO_2 radical formed from ozonolysis with x inserted O_2 in the molecule (beside the peroxy O_2 of the RO_2 radical).

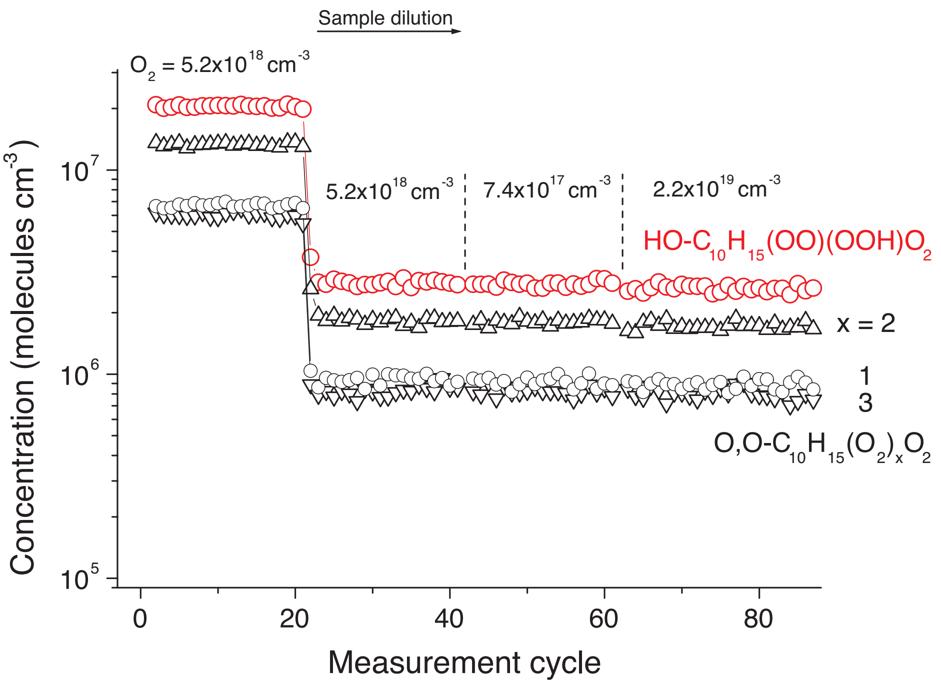

Supplementary Figure 3. O_2 variation in the ionization zone. RO_2 radical measurements from ozonolysis of α-pinene for constant O_2 concentrations (air) in the flow system and varying O_2 concentrations in the ionization region, $[O_3] = 6.1 \times 10^{11}$, $[\alpha\text{-pinene}] = 2.5 \times 10^{12}$ molecules cm^{-3}, reaction time: 7.9 s, using acetate ionization. At measurement cycle 20, the standard sampling tube was replaced by another tube including a dilution unit (dilution: 1/7). Dilution gases were in the order air, N_2 and O_2, resulting in the given O_2 concentrations in the ionization region.

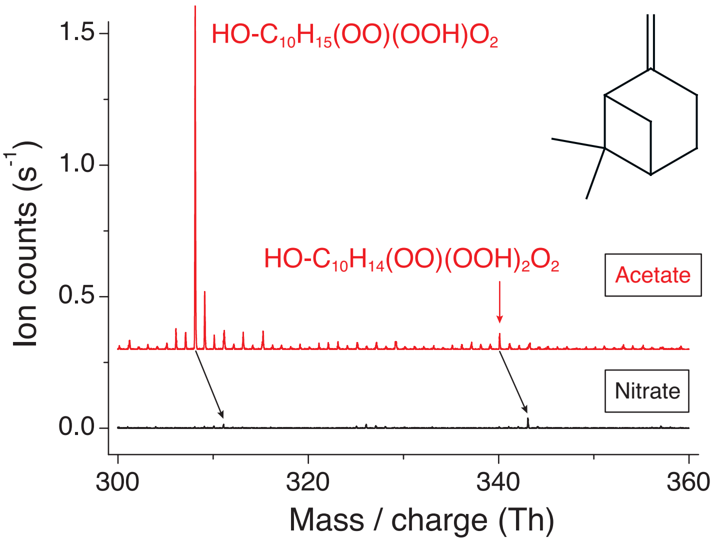

Supplementary Figure 4. Product spectra from OH + β-pinene. Mass spectra recorded for identical reaction conditions from the oxidation of β-pinene using acetate (red) or nitrate (black) for ionization. OH radicals were produced via TME/(β-pinene) ozonolysis. RO_2 radicals appear as adducts with the reagent ions. Signals of nitrate adducts are shifted by three mass units regarding the corresponding acetate adduct signals. The spectra obtained with acetate ionization are offset 0.3 s^{-1} for more clarity. The reaction time was 7.9 s. $[O_3] = 9.1 \times 10^{11}$, $[TME] = 1.0 \times 10^{11}$, and [β-pinene] = 1.05×10^{11} molecules cm^{-3}.

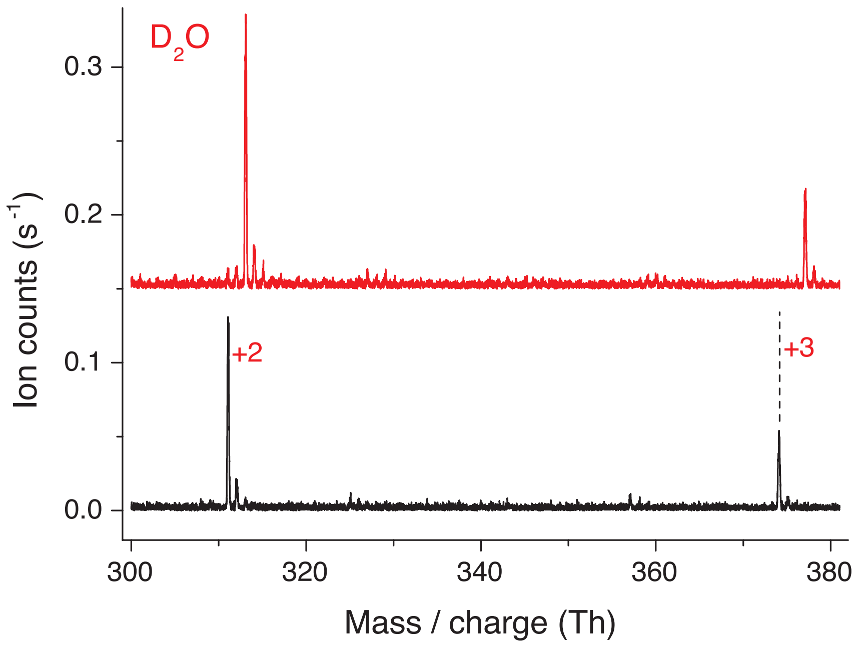

Supplementary Figure 5. H/D exchange experiments. Mass spectra recorded from H_2O_2 photolysis experiments in absence (black) or presence of heavy water (red, $[D_2O] \sim 1.5 \times 10^{17}$ molecules cm^{-3}, [α-pinene] = 5.0×10^{12} molecules cm^{-3}, reaction time: 7.9 s, nitrate ionization. Signal shift (due to H/D exchange of acidic H atoms) by two mass units for HO-$C_{10}H_{16}O_6 \cdot NO_3^-$ at nominal 311 Th and by three nominal mass units for HO-$C_{10}H_{16}O_6 \cdot (HNO_3)NO_3^-$ at nominal 374 Th indicates two acidic H atoms in HO-$C_{10}H_{16}O_6$ leading to the proposed formula **HO**-$C_{10}H_{15}$(OO)(OO**H**)O_2. A comparison of results obtained with dry or humidified air, $[H_2O] \sim 1.5 \times 10^{17}$ molecules cm^{-3}, showed that ′normal′ water did not influence the measured signals.

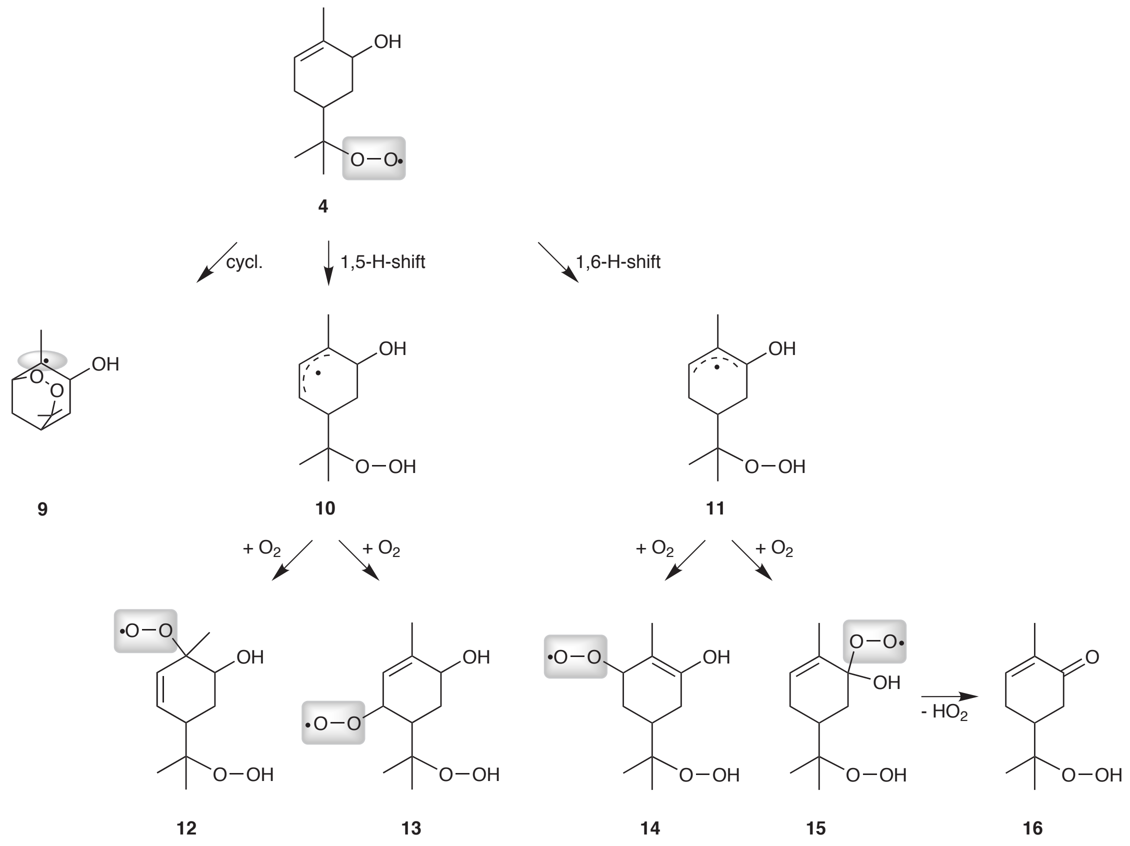

Supplementary Figure 6. Considered reactions of RO_2 radical 4. Proposed reaction scheme for the formation of five O atoms containing RO_2 radicals **12 - 15** starting from **4**.

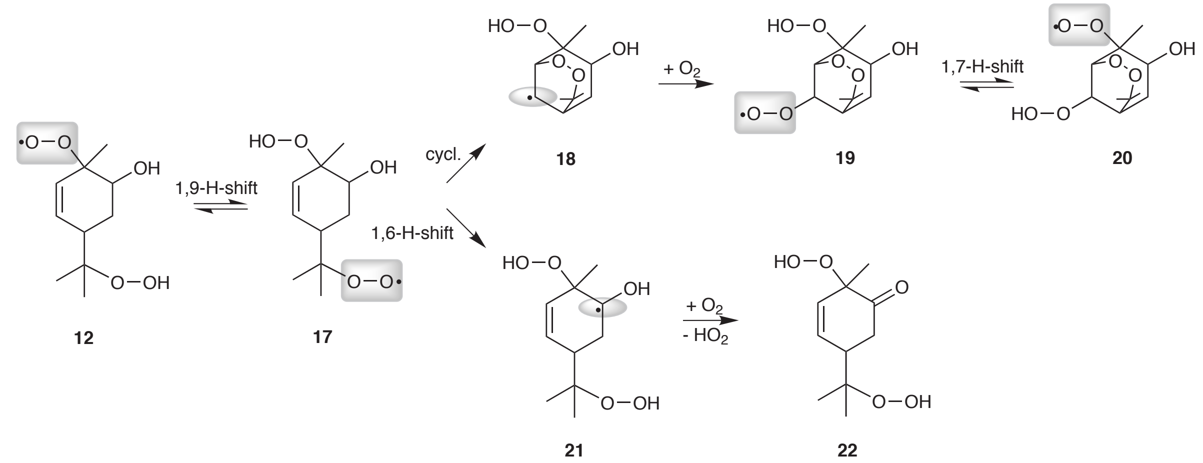

Supplementary Figure 7. Considered reactions of RO_2 radical 12. Proposed reaction scheme for the further reactions of **12** forming the seven O atoms containing RO_2 radical **19**.

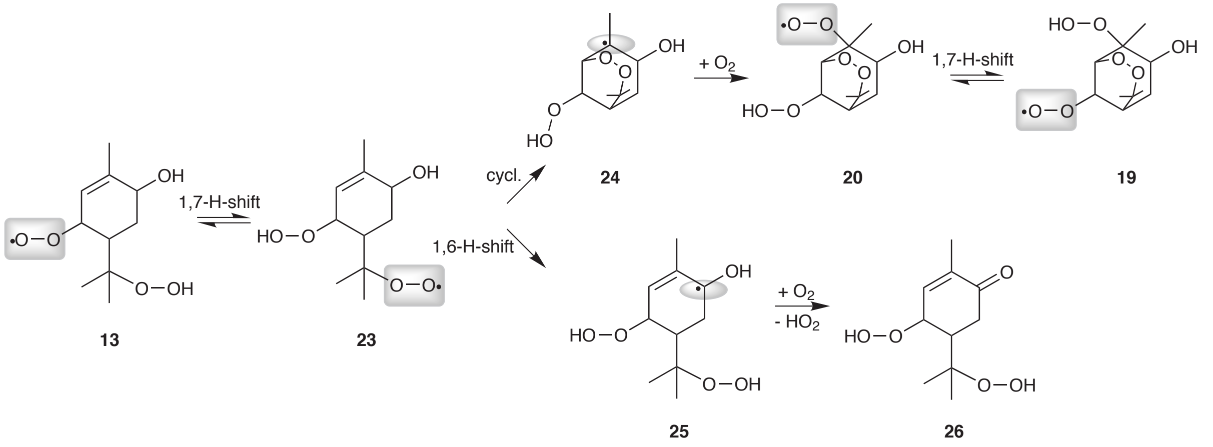

Supplementary Figure 8. Considered reactions of RO_2 radical 13. Proposed reaction scheme for the further reactions of **13** forming the seven O atoms containing RO_2 radical **20**.

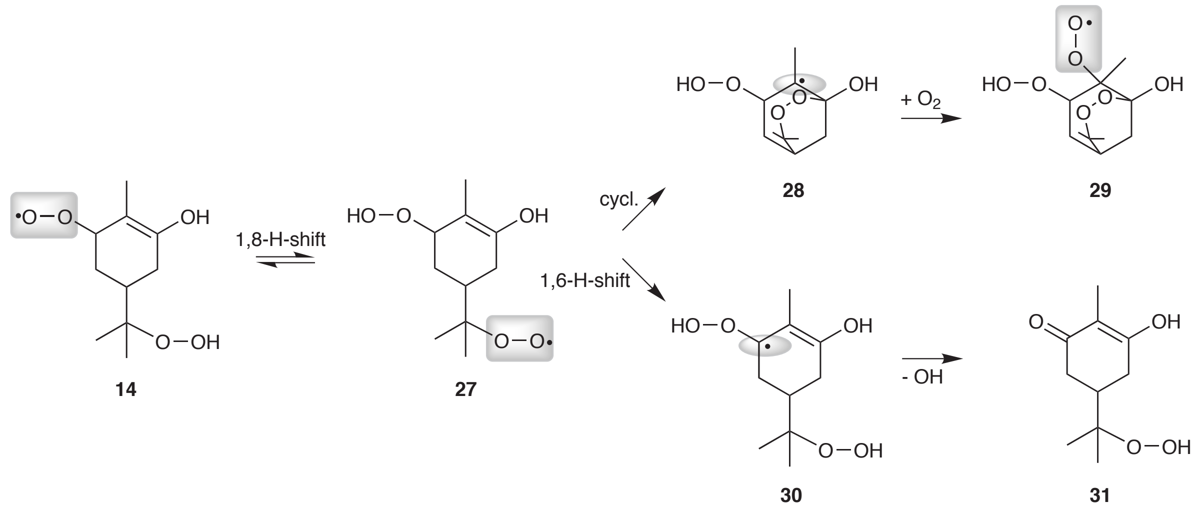

Supplementary Figure 9. Considered reactions of RO_2 radical 14. Proposed reaction scheme for the further reactions of **14** forming the seven O atoms containing RO_2 radical **28**.

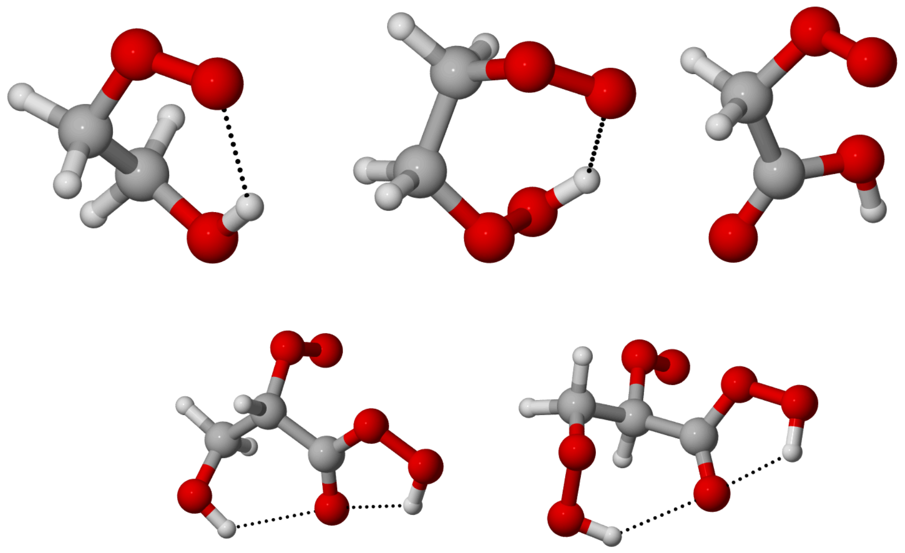

Supplementary Figure 10. Model RO_2 compounds. Lowest-energy conformers of model RO_2 compounds, at the ωB97XD/aug-cc-pVTZ level. Top left: $OOCH_2CH_2OH$. Top middle: $OOCH_2CH_2OOH$. Top right: $OOCH_2C(O)OH$. Bottom left: $HOCH_2CH(OO)C(O)OOH$. Bottom right: $HOOCH_2CH(OO)C(O)OOH$. Colour coding: red=oxygen, gray=carbon, white=hydrogen.

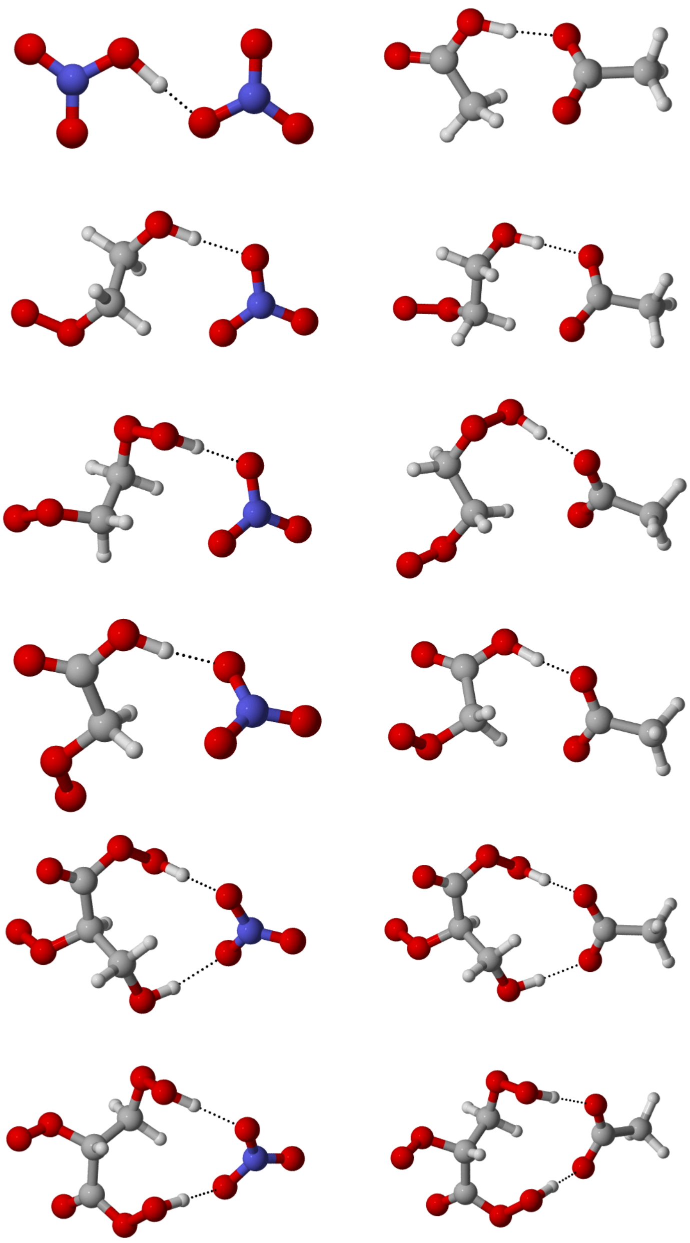

Supplementary Figure 11. Ion-molecule clusters. Lowest-energy structures of various ion-molecule clusters, at the ωB97XD/aug-cc-pVTZ level. Left-hand column, from top to bottom: $HNO_3 \cdot NO_3^-$, $OOCH_2CH_2OH \cdot NO_3^-$, $OOCH_2CH_2OOH \cdot NO_3^-$, $OOCH_2C(O)OH \cdot NO_3^-$, $HOCH_2CH(OO)C(O)OOH \cdot NO_3^-$, $HOOCH_2CH(OO)C(O)OOH \cdot NO_3^-$. Right-hand column, from top to bottom: $CH_3COOH \cdot CH_3COO^-$, $OOCH_2CH_2OH \cdot CH_3COO^-$, $OOCH_2CH_2OOH \cdot CH_3COO^-$, $OOCH_2C(O)OH \cdot CH_3COO^-$, $HOCH_2CH(OO)C(O)OOH \cdot CH_3COO^-$, $HOOCH_2CH(OO)C(O)OOH \cdot CH_3COO^-$. Colour coding: red=oxygen, gray=carbon, blue=nitrogen, white=hydrogen.

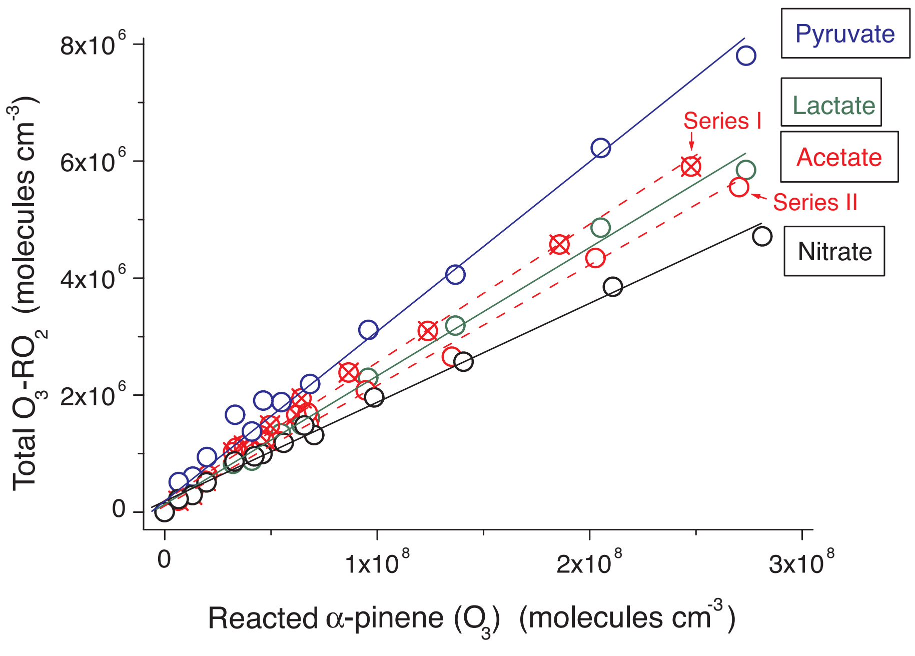

Supplementary Figure 12. Ozonolysis derived RO_2 radicals. Total concentrations of ozonolysis derived RO_2 radicals, O,O-$C_{10}H_{15}(O_2)_xO_2$ with x = 1 - 4, as a function of converted α-pinene using nitrate (black), acetate (red), lactate (olive) or pyruvate (blue) for ionization, $[O_3] = 6.1 \times 10^{11}$, [α-pinene] = $(1.2 - 53) \times 10^{10}$ molecules cm^{-3}, reaction time: 7.9 s. The stated concentrations are estimated values for the different ionization schemes applied. Series I and II from experiments with acetate ionization have been conducted with different acetic acid concentrations in the sheath gas. Only data for a conversion smaller than 3×10^8 molecules cm^{-3} are depicted. The slopes, total RO_2 concentrations vs. reacted α-pinene, give molar formation yields of 2.1 ± 0.1 % (nitrate), 2.7 ± 0.2 % (acetate, series I), 2.1 ± 0.3 % (acetate, series II), 2.4 ± 0.2 % (lactate) and 3.4 ± 0.3 % (pyruvate).

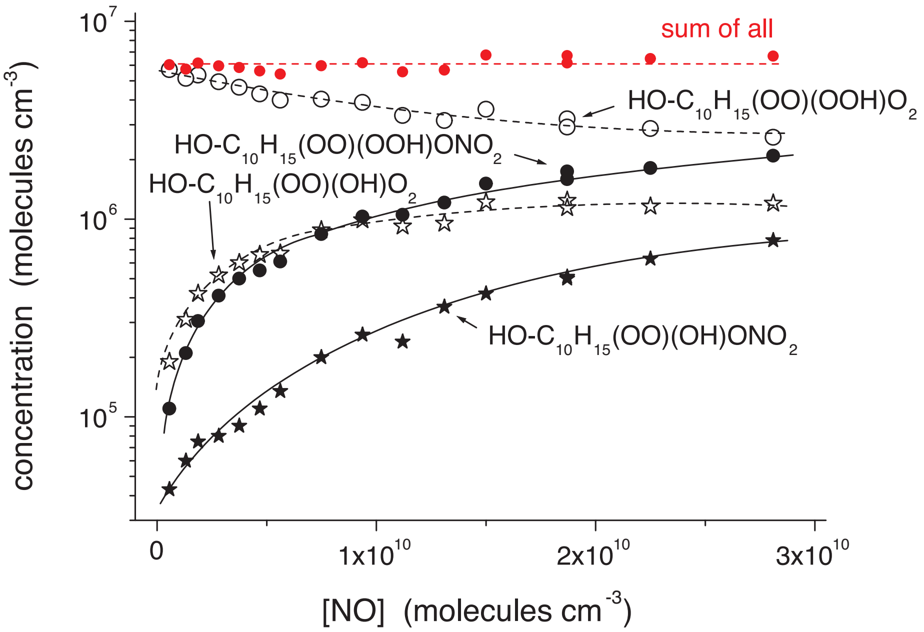

Supplementary Figure 13. Product formation in presence of NO. Highly oxidized organic nitrate formation from the reaction of OH radicals with α-pinene in presence of NO. OH radicals were produced via H_2O_2 photolysis, $[H_2O_2] \sim 1 \times 10^{14}$, [α-pinene] = 5.0×10^{12}, and [NO] = $(5.6 - 280) \times 10^{8}$ molecules cm^{-3}. The reaction time was 7.5 s. RO_2 radicals (open symbol) and organic nitrates (full symbols) were detected as acetate adduct. The red dots show the sum of concentrations of all products including the residual HO-$C_{10}H_{15}(OO)(OOH)O_2$ concentration.

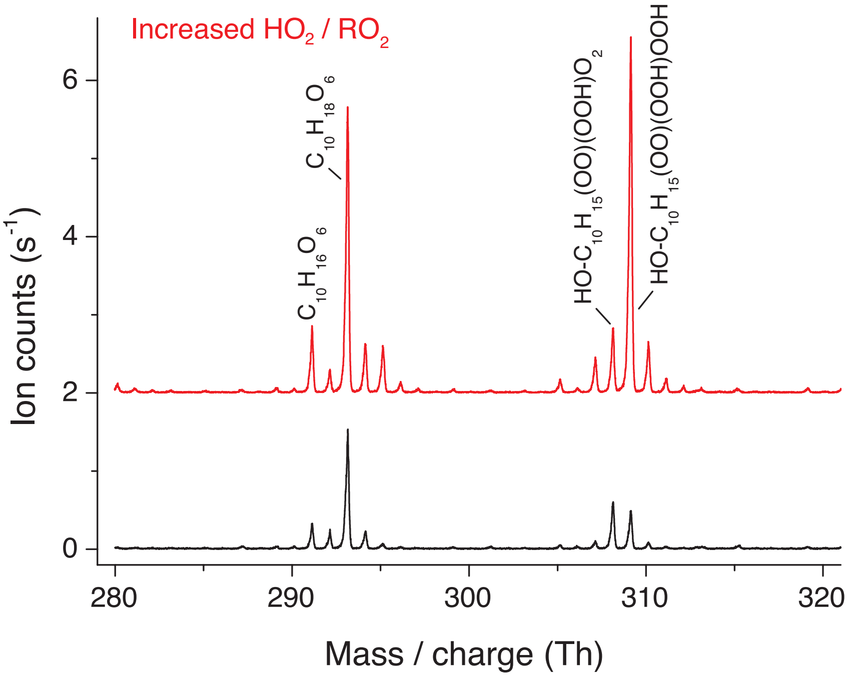

Supplementary Figure 14a. Closed-shell product formation. Recorded mass spectra from H_2O_2 photolysis experiments in the TROPOS flow-tube for reacted α-pinene of ~2 × 10^{10} molecules cm^{-3} (black) and ~4 × 10^{10} molecules cm^{-3} (red); initial α-pinene = 2.0 × 10^{11} molecules cm^{-3}, reaction time: 48 s, sample flow dilution by a factor of 7, acetate ionization. Variation of α-pinene consumption is achieved by variation of the H_2O_2 concentration in the experiment (variation of the gas flow over the H_2O_2 sample). Enhancement of the H_2O_2 concentrations (for constant α-pinene concentrations) leads to an increase of the HO_2 / RO_2 ratio in the system due to the competition reaction OH + H_2O_2 vs. OH + α-pinene. The lack of a reliable detection technique for H_2O_2 does not allow a more quantitative description of the reaction system. The signals with the HOM composition $C_{10}H_{18}O_6$ (proposed HO-$C_{10}H_{15}$(OO)(OOH)OH) is believed to be a reaction product of the HO-$C_{10}H_{15}$(OO)(OOH)O_2 radicals with other RO_2 radicals. With increasing HO_2 in the system, the predominant (detectable) reaction product becomes the hydroperoxide, HO-$C_{10}H_{15}$(OO)(OOH)OOH, see the red spectrum.

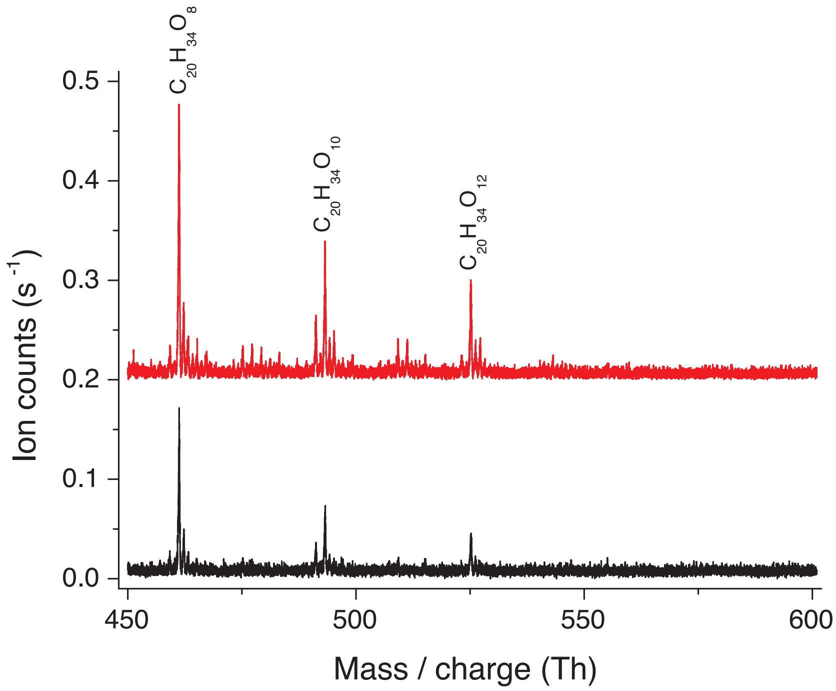

Supplementary Figure 14b. Accretion product formation. Product mass spectra in the range of 450 - 600 Th for identical reaction condition as shown in Supplementary Fig. 14a, reacted α-pinene of ~2 × 10^{10} molecules cm^{-3} (black, lower spectrum) and ~4 × 10^{10} molecules cm^{-3} (red, upper spectrum). The signals detected at nominal 461, 493 and 525 Th are consistent with acetate adducts of $C_{20}H_{34}O_8$, $C_{20}H_{34}O_{10}$ and $C_{20}H_{34}O_{12}$. Their formation can be mechanistically explained via the accretion reaction $RO_2 + R'O_2 \rightarrow ROOR' + O_2$. RO_2 and $R'O_2$ represent peroxy radicals from the OH radical initiated oxidation of α-pinene with three, five or seven O atoms.

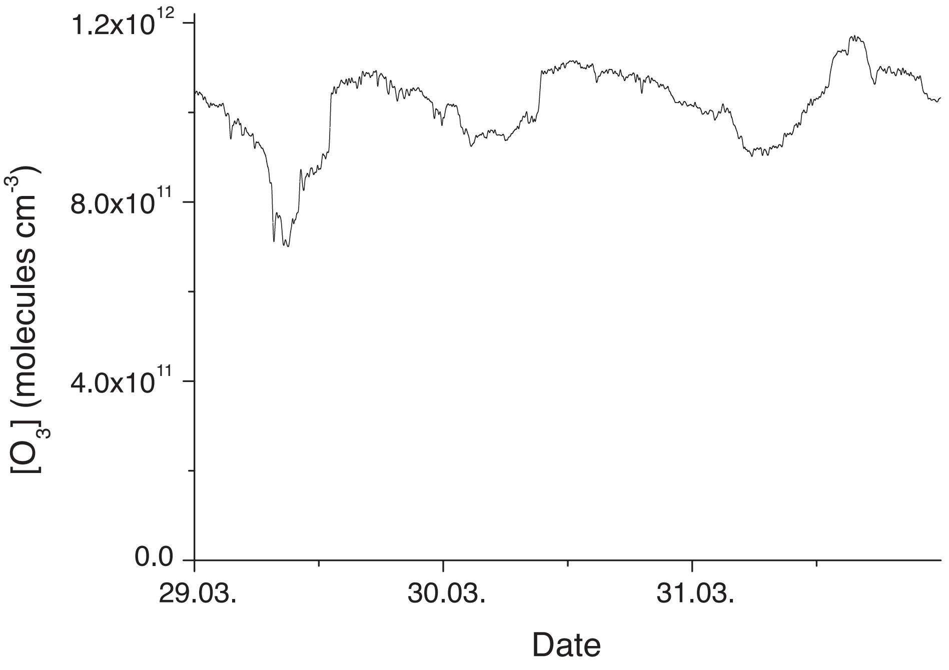

Supplementary Figure 15. Ozone concentration in Hyytiälä. Time series of the ozone concentration measured at the boreal research station SMEAR II, Hyytiälä, Finland, 29.-31.03.2011.

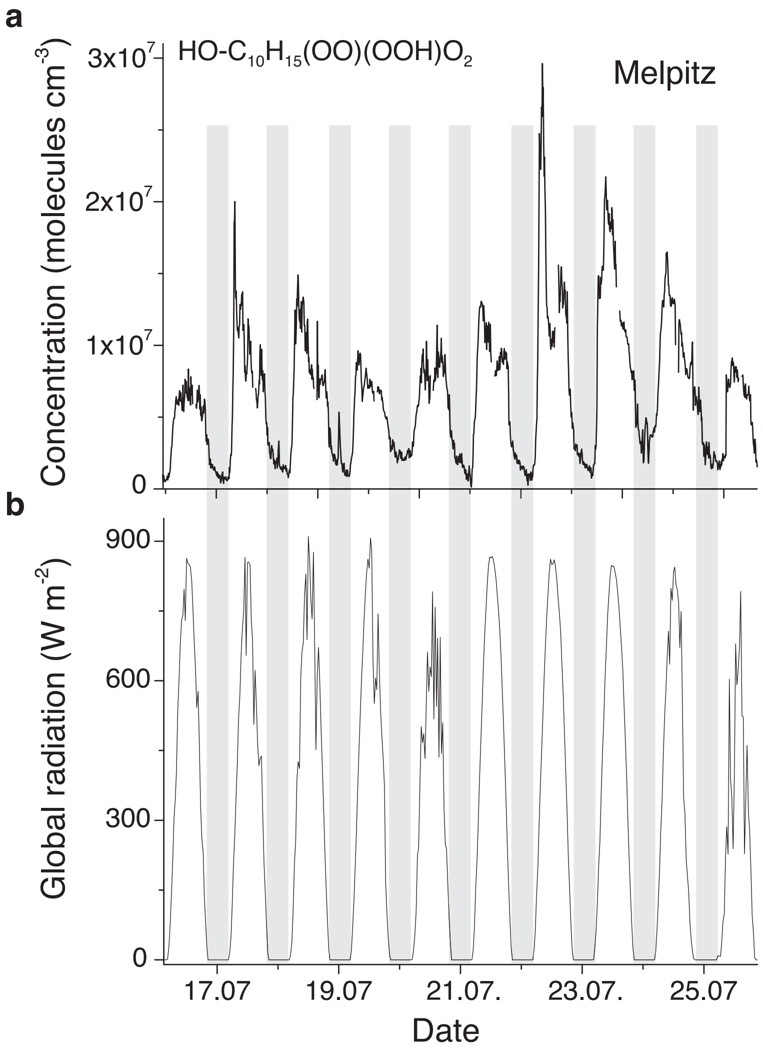

Supplementary Figure 16. Results from the TROPOS research station in Melpitz. Field measurements at the TROPOS research station, Melpitz, Germany, 16.-25.07.2013. a, Time series of the signal attributed to the HO-$C_{10}H_{15}(OO)(OOH)O_2$ radical from the OH radical reaction with terpenes detected by nitrate-CI-APi-TOF measurements. b, Global radiation is taken as a proxy for OH radicals. The HO-$C_{10}H_{15}(OO)(OOH)O_2$ trace follows strictly the diurnal behaviour of the global radiation indicating the link to an OH radical reaction. The given concentrations are the original measurement data using nitrate ionization without a correction by a factor of 40. The relatively large difference of the HO-$C_{10}H_{15}(OO)(OOH)O_2$ radical concentrations comparing Melpitz with Hyytiälä data (Fig. 4a) can be due to a couple of reasons: First, Melpitz is a rural background station whereas Hyytiälä is located in a remote area and the global radiation, connected to the OH radical concentration, in July in Melpitz was much higher than during the Hyytiälä measurements in March. Secondly, it is expected that the monoterpene emission, and consequently the resulting monoterpene concentrations, in the beginning of spring in Hyytiälä were definitely smaller than those in Melpitz in the summertime. That means that both reactant concentrations needed to form the HOM-RO_2 radicals were most likely higher in Melpitz. A more quantitative explanation including an assessment of the loss processes is impossible due to the lack of measurement data.

Supplementary Table 1. Calculated kinetic parameters. Calculated (wB97X-D/aug-cc-pVTZ) parameters for the two possible H-shift and the endo cyclization reaction from RO_2 radical **4** (*anti* conformer).

Structure of TS	E_F[a]	E_R[b]	v_{imag}[c]	κ[d]	k_F[e]	k_R[f]
1,5-H-shift	19.8	18.8	1996i	536	0.66 (65)	2.7
1,6-H-shift	17.7	20.2	1726i	51	0.96 (56)	$1.5x10^{-2}$
endo-cyclization	17.2	13.4	613i	1.5	0.079 (2.7)	$1.4x10^{3}$

[a] Forward barrier (ROO→ QOOH) in kcal mol^{-1}. Calculated with the MC-TST method and in bracket with the B3LYP/6-31+G(d) method.
[b] Reverse barrier (QOOH→ ROO) in kcal mol^{-1}. Calculated with the MC-TST method and in bracket with the B3LYP/6-31+G(d) method.
[c] Imaginary frequency in cm^{-1}.
[d] Eckart quantum tunneling (unitless) on lowest energy conformers.
[e] Rate coefficient for the forward reaction (ROO→QOOH) in s^{-1}
[f] Rate coefficient for the reverse reaction (QOOH→ROO) in s^{-1}

Supplementary Table 2. Calculated kinetic parameters. Calculated (MC-TST/B3LYP) parameters for the possible H-shift reactions from RO_2 radical **20** (*anti* conformer).

Structure of TS	E_F [a]	E_R [b]	v_{imag} [c]	κ [d]	k_F [e]	k_R [f]
1,4-H-shift (H on same C as OH) *syn*	29.9	21.7	1116i	4.8	$2.4x10^{-8}$	$1.1x10^{-2}$
1,4-H-shift (H next to OO bridge) *syn*	39.7	21.0	2131i	7961	$1.3x10^{-13}$	4.6
1,5-H-shift-OH *syn*	23.8	5.0	1450i	7.3	$3.3x10^{-5}$	$1.3x10^{+9}$
1,4-H-shift *anti*	33.8	14.8	2238i	2034	$7.4x10^{-10}$	$9.9x10^{+4}$
1,5-H-shift (H on same C as OOH) *anti*	20.9		1729i	31	$1.4x10^{-2}$	loss of OH
1,5-H-shift-OH *anti*	16.9		952i	1.0	0.79	ring opening

[a] Forward barrier (ROO→ QOOH) in kcal mol^{-1}.
[b] Reverse barrier (QOOH→ ROO) in kcal mol^{-1}.
[c] Imaginary frequency in cm^{-1}.
[d] Eckart quantum tunneling (unitless) on lowest energy conformers.
[e] Rate coefficient for the forward reaction (ROO→QOOH) in s^{-1}
[f] Rate coefficient for the reverse reaction (QOOH→ROO) in s^{-1}

Supplementary Table 3. Calculated kinetic parameters. Calculated (LC-TST/wB97X-D) parameters for the possible H-shift reactions from RO_2 radical **29** (*syn* conformer).

Structure of TS	E_F[a]	v_{imag}[c]	k_F[e]
1,4-H-shift	25.4	2015i	$8.5x10^{-7}$
1,5a-H-shift	26.6	1846i	$7.2x10^{-8}$
1,5b-H-shift	37.4	1990i	$1.3x10^{-15}$
1,4-H-shift	44.0	1950i	$8.5x10^{-21}$

[a] Forward barrier (ROO→ QOOH) in kcal mol^{-1}.
[c] Imaginary frequency in cm^{-1}.
[e] Rate coefficient for the forward reaction (ROO→QOOH) in s^{-1}. Not including tunneling.

Supplementary Table 4. Results from the calculations of the cluster stability. Enthalpies and Gibbs free energies (unit: kcal/mol; at 298.15 K and 1 atm reference pressure) for the formation (from separated molecules and ions) of various ion-molecule clusters, at the ωB97XD/aug-cc-pVTZ level, using the ultrafine integration grid and the Gaussian 09 program suite[19].

Species	ΔH	ΔG
$HNO_3 \cdot NO_3^-$	-29.56	-21.33
$OOCH_2CH_2OH \cdot NO_3$-	-20.71	-12.79
$OOCH_2CH_2OOH \cdot NO_3$-	-22.72	-14.69
$OOCH_2C(O)OH \cdot NO_3$-	-29.66	-20.73
$HOCH_2CH(OO)C(O)OOH \cdot NO_3^-$	-33.33	-23.68
$HOOCH_2CH(OO)C(O)OOH \cdot NO_3^-$	-36.32	-25.95
$CH_3C(O)OH \cdot CH_3COO^-$	-29.26	-17.62
$OOCH_2CH_2OH \cdot CH_3COO^-$	-26.68	-16.52
$OOCH_2CH_2OOH \cdot CH_3COO^-$	-28.89	-18.65
$OOCH_2C(O)OH \cdot CH_3COO^-$	-39.34	-27.46
$HOCH_2CH(OO)C(O)OOH \cdot CH_3COO^-$	-44.35	-32.25
$HOOCH_2CH(OO)C(O)OOH \cdot CH_3COO^-$	-48.46	-36.67

Supplementary Table 5. Estimated vapour pressure of HOMs at 295 K.
The notation of the RO_2 radicals correspond to that used in Fig. 2 and Supplementary Figures 6 - 9.

Substance	SIMPOL.1 $P_{V,}$ (atm)	COSMO-RS $P_{V,}$ (atm)
C_{10} products		
$HO\text{-}C_{10}H_{15}(OO)(OOH)_2$	4.6×10^{-11}	9.9×10^{-11}
$HO\text{-}C_{10}H_{15}(OO)(OOH)ONO_2$	8.2×10^{-11}	8.1×10^{-10}
$HO\text{-}C_{10}H_{15}(OO)(OOH)OH$ *	8.2×10^{-11}	2.0×10^{-10}
C_{20} accretion products ROOR´<br>$RO_2 + R'O_2 \rightarrow ROOR' + O_2$		
$C_{20}H_{34}O_8$<br>$HO\text{-}C_{10}H_{15}(OO)(OOH)\text{-}OO\text{-}C_{10}H_{16}\text{-}OH$<br>$RO_2$: 4; $R'O_2$: 19, 20 or 29	6.4×10^{-16}	
$C_{20}H_{34}O_8$<br>$HO\text{-}C_{10}H_{15}(OO)(OOH)\text{-}OO\text{-}C_{10}H_{16}\text{-}OH$<br>$RO_2$: 3 or 5; $R'O_2$: 19, 20 or 29	8.2×10^{-16}	
$C_{20}H_{34}O_8$<br>$HO\text{-}C_{10}H_{15}(OOH)\text{-}OO\text{-}C_{10}H_{15}(OOH)\text{-}OH$<br>$RO_2$: 12, 13 or 14; $R'O_2$: 12, 13 or 14	8.1×10^{-18}	
$C_{20}H_{34}O_{10}$<br>$HO\text{-}C_{10}H_{15}(OO)(OOH)\text{-}OO\text{-}C_{10}H_{15}(OOH)\text{-}OH$<br>$RO_2$: 12, 13 or 14; $R'O_2$: 19, 20 or 29	2.2×10^{-18}	
$C_{20}H_{34}O_{12}$<br>$HO\text{-}C_{10}H_{15}(OO)(OOH)\text{-}OO\text{-}C_{10}H_{15}(OO)(OOH)\text{-}OH$<br>$RO_2$: 19, 20 or 29; $R'O_2$: 19, 20 or 29;	5.9×10^{-19}	

* proposed molecular formula

Supplementary Notes

1. Influence of unwanted processes in the ionization region

It was checked, whether unwanted processes in the chemical ionization (CI) region (other than ionization) were responsible for the measured signals or influenced them. First, time-dependent RO_2 radical measurements for constant reactant conditions showed a linear behaviour of RO_2 radical concentrations with time providing a clear indication for negligible RO_2 radical generation in the CI region (Supplementary Fig. 1).

Secondly, measurements with varying O_2 concentration in the free-jet flow system (including the CI region) showed distinct O_2-dependent RO_2 radical concentrations obviously caused by competitive processes (unimolecular steps vs. O_2 addition) in the course of RO_2 radical generation[1] (Supplementary Fig. 2). On the other hand, changing O_2 concentrations in the CI region only did not show any effect (Supplementary Fig. 3). These tests confirm that the highly oxidized RO_2 radicals were exclusively formed in the flow system and not in the CI region. Moreover, the time-dependent experiment reveals that the formation of highly oxidized RO_2 radicals in this system proceeds at a time scale of the lowest reaction time of 3 s or less (Supplementary Fig. 1).

2. Theoretical calculation of rate coefficients

2.1 Theory and computational details

The rate coefficients (*k*) of the possible unimolecular H-shift and endo-cyclization reactions have been calculated with Transition State Theory (TST), either using only the lowest energy conformer (LC-TST) or using multiple conformers within a given energy cut-off (MC-TST). The MC-TST rate coefficient is given by[2]:

$$k_{MC-TST} = \kappa \frac{k_B T}{h} \frac{\sum_i \exp\left(-\Delta E_i / k_B T\right) Q_{TS,i}}{\sum_j \exp\left(-\Delta E_j / k_B T\right) Q_{R,j}} \exp\left(-\frac{E_{TS} - E_R}{k_B T} \right), \qquad \text{(S1)}$$

where κ is the Eckart tunnelling correction factor, k_B is the Boltzmann constant, T is the temperature and *h* is Planck's constant. The summations are over all included conformers of the transition state and reactant, respectively. Q_i is the partition function of either transition state (TS) or reactant (R) for each conformer, while ΔE_i is the energy difference between conformer *i* and the lowest energy conformer. E_{TS} and E_R is the lowest energy conformer of the transition state and reactant, respectively. All energies are zero-point (vibrational) energies. To calculate the rate coefficient for the backward reaction, the reactant in Eq.(S1) is substituted with the product (P). For the reactant or product we introduce an energy cut-off in the selection of conformers to include.

We performed systematic conformer searches in Spartan'14 with either the MMFF or SYBYL force fields [3-5]. The searches were run with the keywords (systematic, keepall, keepverbose and geometrycycles = 500). In the MMFF conformer searches of the reactant, product and TS with MMFF were performed with the keyword 'ffhint=Ox~~+0', where *x* is the number of the peroxy oxygen (or C if the radical center is on carbon), MMFF-charge. This

keyword changes the charge of the atom to neutral. The conformer searches of the TS were done by freezing the bond lengths, which changes the most during the reaction. We froze the O–O, O–H and C–O bonds. The length of the frozen bonds were based on a prior optimization with B3LYP/6-31+G(d) of a single TS conformer [6-12].

In the SYBYL searches, the conformer search was followed by a B3LYP/6-31+G(d) single point energy calculation in Gaussian09 and all structures with energy less than 5 kcal/mol was kept for a subsequent B3LYP/6-31+G(d) optimization. The lowest energy structure was then optimized at wB97X-D/aug-cc-pVTZ and frequency calculation performed. Rate coefficients calculated with this approach is called LC-TST/wB97X-D. The Eckart tunneling factor was calculated using the energy of the R, TS and P, and the wB97X-D imaginary frequency.

In the MMFF-charge conformer searches, all conformers found were optimized at the B3LYP/6-31+G(d) level of theory in Gaussian09 and frequencies calculated. The TS conformers were optimized at the same level of theory, with the keywords 'opt=(ts,calcfc,noeigentest)'. MC-TST rate coefficients were then calculated including all these B3LYP/6-31+G(d) conformers, which is called MC-TST/B3LYP. The Eckart tunneling is calculated with the conformers connected from the intrinsic reaction coordinate (IRC) of the lowest energy TS conformer. The electronic structure level is increased to improve the MC-TST/B3LYP rate coefficient. However, to limit the computational time an energy cut-off, at 2 kcal/mol in the B3LYP/6-31+G(d) energies is implemented. The remaining conformers are then optimized at the wB97X-D/aug-cc-pVTZ level and frequencies calculated. The Eckart tunneling is calculated with the R and P conformers connected via the B3LYP/IRC of the lowest energy wB97X-D optimized TS conformer. Energy and frequencies of these conformers are calculated with the wB97X-D/aug-cc-pVTZ method. We call this approach MC-TST/wB97XD.[13]

Clearly the MC-TST/wB97XD is the best approach as it included both high level conformational sampling and high level energetics and we used this for the first reaction step (the three oxygen compound **4**). This was not feasible for the subsequent reactions but we tested high-level conformational sampling via MC-TST/B3LYP and the high level energetics via LC-TST/wB97X-D and although both might lead to high H-shift rate coefficients, they should provide order of magnitude rate coefficients.

2.2 Calculated rate coefficients

Compound 4, Supplementary Fig.6, exist in both a syn and anti conformer. We have not attempted to run calculations on all possible combination in all of the steps, but focus on showing that it is possible to react within the timeframe of the experiment from compound **4** to a seven oxygen containing RO_2 radical with a reasonable lifetime. The first possible steps in the reaction of **4** are shown in Supplementary Fig. 6, and the calculated rate coefficients for two H-shifts and the endo-cyclization are given Supplementary Table 1. We find that the forward reaction of the initial H-shift reactions happens with a rate coefficient of about 1 s^{-1}, and is about an order of magnitude faster than the cyclization reaction. The 1,6-H-shift (*anti* only) and endo cyclization (*syn* and *anti*) reactions were also considered previously at the B3LYP level.[14] The previously calculated B3LYP rate coefficients (faster than 11.5 s^{-1} for the 1,6-H-shift and 2.6/0.6 s^{-1} for the *syn*/*anti* endo-cyclization reactions) are in agreement with our B3LYP rate coefficients shown in brackets in Supplementary Table 1.

Following the H-shift reactions, rapid O_2 addition on the timescale of 10^7 s^{-1} will lead to the five oxygen containing RO_2 radicals **12-15**, shown in Supplementary Fig. 6. Compound **15** would likely eliminate HO_2 and is not considered further. In Supplementary Figures 7 - 9, we show possible reaction mechanisms RO_2 radicals **12-14**. Some *syn*/*anti* conformers of compounds **12-14** will be able to undergo OO-HOO H-shift, which were recently shown to be

very rapid, partly due to large tunneling factors.[15] Here, we tested one of the OO-HOO H-shifts and found with the LC-TST/wB97X-D method a forward rate coefficient of about 300 s^{-1} for the OO-HOO, 1,8-H-shift from **14** to **27**. The OO-HOO H-shift rate coefficients are possibly a bit faster in 1,7-H-Shift and a bit slower in the 1,9-H.shift.[15]

The generated five oxygen containing RO_2 radicals, **17**, **23**, and **27** have a range of possible H-shifts, one of which is the 1,6-H-shift, shown in Supplementary Figures 7 - 9. The rate coefficients of H-shifts in RO_2 radicals are often fastest for 1,5 and 1,6 H-shifts and often enhanced further with OH or OOH group attached to the C from which the H is abstracted.[16] The H-shift examples shown in Supplementary Figures 7 - 9 all lead to termination via loss of OH (if abstraction from C with OOH attached, compounds **30**/**31**) or loss of HO_2 after O_2 addition (if abstraction from C with OH attached, compound **21**/**22** or **25**/**26**). We have not calculated the rate coefficients of these H-shifts possibilities, but expect them to be of the order of 1 s^{-1}.[15,16]

In addition, the RO_2 radicals **17**, **23**, and **27** have the possibility of endo-cyclization similar to compound **4** as illustrated in Supplementary Figures 7 - 9. We have used the MC-TST/B3LYP method to calculate the rate coefficients for endo-cyclization in both the *anti* and *syn* for compound **23** and get rate coefficients of 1.3 s^{-1} and 6.8 s^{-1}, respectively. With LC-TST/wB97X-D we find for the *syn* conformer of **27** an endo-cyclization rate coefficient of 0.13 s^{-1}. These rate coefficients are likely competitive with the possible H-shift reactions and after subsequent O_2 addition lead to a seven oxygen containing RO_2 radical. The compounds **19** and **20**, from Supplementary Figures 7 and 8, respectively can interconvert via a rapid OO-HOO H-shift.

Based on these rate coefficients it seems plausible that the seven oxygen containing RO_2 radicals **19**, **20** and **29** will be formed in reasonable amounts within the timescale of the experiment (seconds), assuming that these seven oxygen containing RO_2 radicals do not further react rapidly.

To investigate the lifetime of this RO_2, we have calculated the rate coefficients for the H-shift reactions of the RO_2 radicals **20** and **29**. The rate coefficients are calculated with MC-TST/B3LYP (**20**) and LC-TST/wB97X-D (**29**) methods, and are given in Supplementary Tables 2 and 3, respectively. Only forward rates have been calculated for **29**. It is clear from the tables that the only reaction that might have an impact is the abstraction of the hydrogen on the OH group via an OO-HO 1,5-H-shift, which has a MC-TST/B3LYP rate coefficient of 0.79 s^{-1}. However, a significantly higher level calculation (LC-TST/F12/wB97X-D/aug-cc-pVTZ) on a similar OO-HO 1,5-H-shift found a rate coefficient of about 10^{-3} s^{-1}.[15] Thus, the rate coefficient 0.79 s^{-1} based on MC-TST/B3LYP calculations represents most likely an overestimation. We find it likely that these seven oxygen containing RO_2 radicals **19**, **20** and **29** will have lifetimes of at least few 10 seconds in the experiment.

3. Theoretical calculations on the cluster stability, nitrate vs. acetate

3.1 Computational details

Configurational sampling was carried out using an approach similar to that described in Rissanen et al.[17]. All possible conformers of each molecule or ion-molecule cluster were first generated by scanning over torsional angles at 120° intervals using the MMFF force field and the Spartan ’14 program[3]. For the ion-molecule clusters, the conformer search was initiated from a structure containing the maximum number of intermolecular hydrogen bonds (1 or 2 depending on the molecule). In the force-field calculations, the charge of the RO_2 radical oxygen was manually set to zero using the FFHINT keyword, as the default atom type assignment led to a negative charge on this group. The MMFF conformer generation was

followed by ωB97XD/6-31+G(d)[18] single-point energy evaluations on all conformers, and subsequent ωB97XD/6-31+G(d) optimizations on structures with ωB97X/6-31+G(d) single-point energies within 5 kcal/mol of the lowest-energy conformer. The lowest-energy structure for each molecule and cluster was then selected for a subsequent higher-level optimization and frequency calculation at the ωB97XD/aug-cc-pVTZ level using the Gaussian 09 program suite[19], with the ultrafine integration grid. Default convergence criteria were used, except when these led to spurious low imaginary frequencies; in these cases the tight optimization criteria were applied, leading to negligible changes in energy but the disappearance of the imaginary frequencies. The nitrate ion was constrained to have the experimentally observed D3h symmetry. (Enforcing symmetry has a negligible effect on the energy and enthalpy, but a significant effect on the rotational entropy.) Formation enthalpies and free energies were computed using the standard rigid rotor and harmonic oscillator models.

3.2 Calculated cluster stabilities

The greater sensitivity of acetate (CH_3COO^-) chemical ionization mass spectrometry (CIMS) compared to nitrate (NO_3^-) CIMS, and the greater difference between the sensitivities toward OH oxidation products compared to O_3 oxidation products, may be explained by a combination of several factors. First, since nitric acid is a much stronger acid than acetic acid, the nitrate ion is a weaker base than the acetate ion. Thus, acetate ions should in general bind more strongly to acidic organic groups (such as alcohols, peroxides, carboxylic acids or peroxy acids). Second, the nitric acid – nitrate dimer is somewhat more strongly bound than the acetic acid - acetate dimer. Ligand exchange reactions of the type $HA \cdot A^- + X \Rightarrow X \cdot A^- + HA$ (which form the dominant charging mechanism in these CIMS setups) are thus generally much more favourable for a given molecule X when $A^- = CH_3COO^-$ than when $A = NO_3^-$, since the reactant cluster is weaker and the product cluster stronger. Thus, the greater sensitivity of acetate CIMS in general is easily understandable based on the chemical properties of the reagent ions. However, the large difference in relative sensitivities toward OH and O_3 oxidation products is more challenging to explain. One possibility is that the greater basicity of the acetate ion makes it more sensitive toward the relative acidity, or more generally the relative H-bond donor strength, of organic H-bond donor groups. For example, the relative sensitivity of acetate CIMS toward carboxylic acid groups could thus be expected to be greater than that of nitrate CIMS.

In order to investigate the validity of these explanations, and further understand the observed differences in sensitivity between acetate - and nitrate - based detection schemes, calculations were performed on a series of model peroxy radical (RO_2) species with either one or two functional groups in addition to the COO radical group. The chosen functional groups were alcohol (OH), hydroperoxide (OOH), carboxylic acid (C(O)OH) and peroxy acid groups (C(O)OOH), since these are believed to be the main functional groups produced by autoxidation processes. The structures of the model RO_2 compounds investigated are shown in Supplementary Fig. 10.

The structures of the clusters of these five RO_2 model compounds with the two charger ions are shown in Supplementary Fig. 11 (along with the nitric acid – nitrate and acetic acid – acetate clusters). The binding enthalpies and free energies of all clusters are given in Supplementary Table 4.

The results in Supplementary Table 4 support the general hypothesis that acetate is more strongly bound to the different RO_2 species than nitrate is, while simultaneously the binding of $CH_3C(O)OH \cdot CH_3COO^-$ is somewhat weaker than that of $HNO_3 \cdot NO_3^-$. An interesting detail is that while the formation enthalpies of the $CH_3C(O)OH \cdot CH_3COO^-$ and $HNO_3 \cdot NO_3^-$ clusters are almost identical, the formation free energies differ by almost 4 kcal/mol. This is mostly due to the relative entropies of the free ions: the nitrate ion has a

low entropy due to its high rotational symmetry number (six) and high vibrational frequencies, while the acetate ion has no rotational symmetry and one very low-frequency vibration corresponding to the torsional motion of the methyl group, leading to a high entropy. (Treating the methyl torsion of the acetate ion as a hindered rotation using the HinderedRotor package of the Gaussian program[20] does not significantly change the free energy of the acetate ion, and thus the formation free energy of the $CH_3C(O)OH{\cdot}CH_3COO^-$ cluster, as the entropy decrease at 298.15 K is almost exactly matched by a decrease in the vibrational zero-point energy.)

For the $RO_2{\cdot}NO_3^-$ clusters, the computed energetics in Supplementary Table 4 match the patterns predicted for closed-shell $ELVOC{\cdot}NO_3^-$ clusters by Hyttinen et al.[21]. Organic molecules or radicals with only one (OH, OOH or even C(O)OH) H-bond donating functional group do not bind to NO_3^- strongly enough to compete with HNO_3, and are therefore not detectable using nitrate CIMS. Even for the model RO_2 radicals with two functional groups investigated here, the $HNO_3{\cdot}NO_3^- + RO_2 => RO_2{\cdot}NO_3^- + HNO_3$ ligand exchange reactions are only favourable by a few kcal/mol with respect to the free energy. Due to steric constraints in the hydrogen bonding patterns, the $RO_2{\cdot}NO_3^-$ clusters of some of the larger RO_2 formed in autoxidation might well be a few kcal/mol less strongly bound than those studied here. This would lead to low detection efficiencies with nitrate CIMS despite the presence of multiple H-bonding groups – as predicted for the speculative sterically hindered $C_6H_8O_8$ cyclohexene autoxidation product by Hyttinen et al.[21]. O_3 oxidation has a greater probability than OH oxidation of opening up the carbon backbone of endocyclic alkenes, thus reducing steric hindrances for H-bonding of the products. It could therefore be speculated that OH – initiated autoxidation leads, on average, to RO_2 species with less hydrogen bonding flexibility, and thus lower nitrate CIMS detection efficiencies, than O_3 – initiated autoxidation. This is clearly the case for the bicyclic RO_2 compound proposed in Fig. 2. Furthermore, Supplementary Table 4 shows that RO_2 species containing peroxide groups bind somewhat more strongly to nitrate than equivalent RO_2 radicals with hydroxyl groups. This somewhat surprising observation is likely related to the presence of intramolecular hydrogen bonds already in the isolated RO_2 radicals (Supplementary Fig. 10). The greater H-bonding ability of OH groups compared to OOH groups is thus cancelled out, as both the reactants and the products of the clustering reaction contain COH…O hydrogen bonds. For a given number of oxygen atoms, OH – initiated autoxidation products of alkenes are very likely to contain at least one more OH group than the O_3 – initiated autoxidation products. Based on the results of Supplementary Table 4, this will also lead to a weaker binding to nitrate, and thus a lower detection efficiency with nitrate CIMS.

The $RO_2{\cdot}CH_3COO^-$ clusters are all more strongly bound than the corresponding $RO_2{\cdot}NO_3^-$ clusters, both in an absolute sense, and relative to the neutral acid-ion cluster. Even the presence of a single peroxide or carboxylic acid group is enough to make the binding of a RO_2 radical to acetate competitive with that of acetic acid. This explains why acetate CIMS is highly effective at detecting products of both OH- and O_3-initiated autoxidation. The binding of RO_2 radicals with two H-bond donating functional groups to CH_3COO^- is more than 10 kcal/mol stronger than the binding of acetic acid to CH_3COO^-. Thus, acetic acid is not able to compete with the multiply substituted RO_2 at any reasonable concentration ratio, explaining the lack of dependence of the detection efficiency of autoxidation products on the acetic acid concentration, see results in Fig. 3. As expected, the relative sensitivity of acetate CIMS to carboxylic acid groups compared to OH or OOH groups is also much larger than that of nitrate CIMS. If OH – initiated autoxidation has a larger probability of forming carboxylic acid groups than O_3 – initiated autoxidation, this may also help explain the differences in relative sensitivities toward the two groups of products.

4. Product formation from the reaction of OH radicals with α-pinene in presence of NO

Supplementary Fig. 13 shows the results of the HOM formation from the reaction of OH radicals with α-pinene in presence of NO. NO additions were varied in in the range of $(5.6 - 280) \times 10^8$ molecules cm^{-3}. The red dots show the sum of concentrations of all products arising from the reaction of HO-$C_{10}H_{15}(OO)(OOH)O_2$ radicals with NO including the residual HO-$C_{10}H_{15}(OO)(OOH)O_2$ concentration. This summation yielded an almost constant value for the whole range of NO additions. Thus, the total product amount seem to be in accordance with the amount of reacted HO-$C_{10}H_{15}(OO)(OOH)O_2$ radicals. Furthermore, that indicates that the HO-$C_{10}H_{15}(OO)(OOH)O_2$ radical formation was not significantly influenced by the NO additions, even for the highest NO concentration of 2.8×10^{10} molecules cm^{-3}. Consequently, the RO_2 isomerization steps leading to HO-$C_{10}H_{15}(OO)(OOH)O_2$ must be faster than the corresponding RO_2 reactions with NO.

4.1 Possible mechanistic explanation

With increasing NO concentrations, increasing signals with the composition $C_{10}H_{17}O_8N$ and $C_{10}H_{17}O_6$ appeared with relatively high signal strength, and with lower intensity a signal for $C_{10}H_{17}O_7N$. The signal of $C_{10}H_{17}O_8N$ can be attributed to the corresponding organic nitrate, HO-$C_{10}H_{15}(OO)(OOH)ONO_2$ (full circle), starting from the HO-$C_{10}H_{15}(OO)(OOH)O_2$ radical (open circle) via $RO_2 + NO \rightarrow RONO_2$:

$$\text{HO-}C_{10}H_{15}(OO)(OOH)O_2 \quad + \ NO \ \rightarrow \quad \text{HO-}C_{10}H_{15}(OO)(OOH)ONO_2 \qquad (8)$$

The signal of $C_{10}H_{17}O_6$ can be formally assumed to be an alkoxy radical formed via RO_2 + $NO \rightarrow RO + NO_2$:

$$\text{HO-}C_{10}H_{15}(OO)(OOH)O_2 \quad + \ NO \ \rightarrow \quad \text{HO-}C_{10}H_{15}(OO)(OOH)O \ + \ NO_2 \qquad (9)$$

It is unlikely that the detection of alkoxy radicals (RO) is successful in our system because of the expected low RO radical lifetime. Thus, it can be speculated that the RO radical undergoes a rapid internal H atom transfer from the OOH group forming a new RO_2 radical with an additional OH group, i.e. HO-$C_{10}H_{15}(OO)(OH)O_2$ (open star in Supplementary Fig. 13):

$$\text{HO-}C_{10}H_{15}(OO)(OOH)O \quad \rightarrow \quad \text{HO-}C_{10}H_{15}(OO)(OH)O_2 \qquad (10)$$

The detection of the subsequently formed organic nitrate, HO-$C_{10}H_{15}(OO)(OH)ONO_2$ (full star in Supplementary Fig. 13), from this new RO_2 radical supports this idea.

$$\text{HO-}C_{10}H_{15}(OO)(OH)O_2 \quad + \ NO \ \rightarrow \quad \text{HO-}C_{10}H_{15}(OO)(OH)ONO_2 \qquad (11)$$

5. Estimated vapour pressure of HOMs

The vapour pressures of three relevant closed-shell HOMs formed from the reaction of HO-$C_{10}H_{15}(OO)(OOH)O_2$ with either HO_2 (HO-$C_{10}H_{15}(OO)(OOH)_2$), NO (HO-$C_{10}H_{15}(OO)(OOH)ONO_2$) or other RO_2 radicals (proposed structure: HO-$C_{10}H_{15}(OO)(OOH)OH$) as well as for the C_{20} accretion products $C_{20}H_{34}O_8$, $C_{20}H_{34}O_{10}$ and $C_{20}H_{34}O_{12}$ formed via $RO_2 + R'O_2 \rightarrow ROOR' + O_2$ were calculated based on the group contribution method SIMPOL.1 by Pankow and Asher[22]. In the case of $C_{20}H_{34}O_8$, three different combinations for $RO_2 + R'O_2$ have been considered. SIMPOL.1 states group contributions for all needed functional groups of these molecules except that for the endoperoxide moiety. Therefore, the increment of an aliphatic/cyclic ether was taken as a proxy for the endoperoxide moiety. The use of this proxy overestimates the calculated vapour pressures slightly. The calculated vapour pressures applying SIMPOL.1[22] are all below 10^{-10} atm, and thus of the order of magnitude as the vapour pressure of mixed H_2SO_4-H_2O-$(NH_4)_2SO_4$ solutions[23], whose value varies depending on the ionic ratio and the relative humidity.

Vapour pressures were also estimated for the same closed-shell HOMs deduced from the RO_2 radical **29** using the COSMO-RS approach[24], as implemented in the COSMOtherm program[25]. In this approach, charge density surfaces are first computed via quantum chemical methods. The charge density surfaces can then be used to model intermolecular interactions in a computationally affordable manner. Finally, statistical thermodynamics methodology is used to compute chemical potential differences, from which vapour pressures may be determined. While not quantitatively accurate for saturation vapour pressures, this approach has the advantage of needing no system-specific parameterizations, and treating (albeit in an approximate way) the real chemical interactions present in the real chemical systems. Conformers of the three studied structures (with the NO product assumed to have rearranged to the low-energy structure HO-$C_{10}H_{15}(OO)(OOH)ONO_2$ rather than HO-$C_{10}H_{15}(OO)(OOH)OONO$ were first generated using the systematic conformer search in Spartan'14 [3], with subsequent B3LYP/6-31+G(d) calculations to eliminate high-energy conformers as described in Rissanen et al.[17]. The 20 lowest-energy conformers (with different H-bonding patterns and thus potentially different charge density surfaces) from the B3LYP optimizations were then selected for the COSMO-RS and gas-phase calculations on Turbomole[26], using the standard and default BP/TZVP method to generate the input files for the COSMO vapour pressure calculations.

Both sets of calculations show a reasonable agreement with exception of the organic nitrate (difference by a factor of 10), see Supplementary Table 5. Nevertheless, these closed-shell HOMs can be treated as low-volatile and polar substances condensing easily on existing surfaces.

Supplementary References

1. Jokinen, T. *et al.* Rapid autoxidation forms highly oxidized RO_2 radicals in the atmosphere. *Angew. Chem. Int. Ed.* **53,** 14596-14600 (2014).
2. Vereecken, L. & Peeters, J. The 1,5-H-shift in 1-butoxy: A case study in the rigorous implementation of transition state theory for a multirotamer system. *J. Chem. Phys.*, **119,** 5159-5170 (2003).
3. Spartan'14; Wavefunction Inc. Irvine, CA (2014).
4. Halgren, T. A. Merck molecular force field. I. Basis, form, scope, parameterization, and performance of MMFF94. *J. Comput. Chem.*, **17,** 490-519 (1996).
5. Clark, M., Cramer, R. D. & Van Opdenbosch, N. Validation of the general purpose tripos 5.2 force field. *J. Comput. Chem.*, **10,** 982-1012 (1989).
6. Shao, Y. *et al.* Advances in methods and algorithms in a modern quantum chemistry program package. *Phys. Chem. Chem. Phys.*, **8,** 3172-3191 (2006).
7. Becke, A. D. Density-functional thermochemistry. III. The role of exact exchange. *J. Chem. Phys.*, **98,** 5648-5652 (1993).
8. Lee, C., Yang, W. & Parr, R. G. Development of the Colle-Salvetti correlation-energy formula into a functional of the electron density. *Phys. Rev. B*, **37,** 785-789 (1988).
9. Ditchfield, R., Hehre, W. J. & Pople, J. A. Self-consistent molecular - orbital methods. IX. An extended gaussian-type basis for molecular- orbital studies of organic molecules. *J. Chem. Phys.*, **54,** 724-728 (1971).
10. Clark, T., Chandrasekhar, J., Spitznagel, G. W. & Schleyer, P. v. R. Efficient diffuse function-augmented basis sets for anion calculations. III. The 3-21+G basis set for first-row elements, Li–F. *J. Comput. Chem.*, **4,** 294-301 (1983).
11. Frisch, M. J., Pople, J. A. & Binkley, J. S. Self-consistent molecular orbital methods 25. Supplementary functions for Gaussian basis sets. *J. Chem. Phys.*, **80,** 3265-3269 (1984).
12. Frisch, M. J. *et al.* Gaussian 09 Revision D.01. Gaussian Inc. Wallingford CT 2009.
13. Møller, K. H., Otkjær, R. V., Hyttinen, N., Kurten, T. & Kjaergaard, H. G. unpublished.
14. Vereecken, L., Muller, J.-F. & Peeters, J. Low-volatility poly-oxygenates in the OH-initialed atmospheric oxidation of a-pinene: impact of non-traditional peroxyl radical chemistry. *Phys. Chem. Chem. Phys.*, **9,** 5241-5248 (2007).
15. Jørgensen, S. *et al.* Rapid hydrogen shift scrambling in hydroperoxy-substituted organic peroxy radicals. *J. Phys. Chem. A* **120,** 266-275 (2016).
16. Crounse, J. D., Nielsen, L. B., Jørgensen, S., Kjaergaard, H. G. & Wennberg, P. O. Autoxidation of organic compounds in the atmosphere. *J. Phys. Chem. Lett.* **4,** 3513-3520 (2013).

17. Rissanen, M. P. *et al.* The formation of highly oxidized multifunctional products in the ozonolysis of cyclohexene. *J. Am. Chem. Soc.* **136,** 15596-15606 (2014).

18. Chai, J.-D. & Head-Gordon, M. Long-range corrected hybrid density functionals with damped atom–atom dispersion corrections. *Phys. Chem. Chem. Phys.* **10,** 6615-6620 (2008).

19. Frisch, M. J. *et al.* Gaussian 09, revision C.01 and D.01, Gaussian Inc.: Wallingford, CT (2009).

20. Ayala, P. Y. & Schlegel, H. B. Identification and treatment of internal rotation in normal mode vibrational analysis. *J. Chem. Phys.* **108,** 2314-2325 (1998).

21. Hyttinen, H. *et al.* Modeling the detection of highly oxidized cyclohexene ozonolysis products using nitrate-based chemical ionization. *J. Phys. Chem. A* **119,** 6339-6345 (2015).

22. Pankow, J. F. & Asher, W. E. SIMPOL.1: a simple group contribution method for predicting vapor pressures and enthalpies of vaporization of multifunctional organic compounds. *Atmos. Phys. Chem.* **8,** 2773-2796 (2008).

23. Marti, J. J. *et al.* H_2SO_4 vapor pressure of sulfuric acid and ammonium sulfate solutions. *J. Geophys. Res.* **102,** 3725-3735 (1997).

24. Eckert, F. & Klamt, A. Fast solvent screening via quantum chemistry: COSMO-RS approach. *AIChE Journal* **48***,* 369-385 (2002).

25. Eckert, F. & Klamt, A. COSMOtherm, Version C3.0, Release 15.01; COSMOlogic GmbH & Co. KG, Leverkusen, Germany, 2014.

26. TURBOMOLE V7.0 2015, a development of the University of Karlsruhe and the Forschungszentrum Karlsruhe GmbH, 1989-2007, TURBOMOLE GmbH, since 2007, available from http://www.turbomole.com.
