# Supplementary material for: Hydroxyl radical-induced formation of highly oxidized organic compounds
Source: Nat Commun. 2016 Dec 2;7:13677. doi: 10.1038/ncomms13677 (PMC5146283; doi:10.1038/ncomms13677)
Supplement: Peer Review File [file ncomms13677-s2.pdf]

Reviewers' comments:

Reviewer #1 (Remarks to the Author):

This manuscript uses a combination of laboratory and field measurements to demonstrate that OH-initiated oxidation is a substantial source of low-volatility organic compounds and that this pathway must be considered in models of organic aerosol formation in the troposphere. The key insight is that the ion used in chemical ionization mass spectrometry can show marked differences in sensitivity to differently functionalized species, and the authors therefore introduce a suite of ionizing methods to analyze the oxidation process, and compare pure OH-initiation with the combined OH and O3 initiation inherent in ozonolysis experiments. This is a very thorough set of research, and despite the complexity of the oxidation systems the important conclusions about the importance of OH-initiation and about the likely mechanism of oxidation are in my view robust.

Let's look at the these conclusions in turn. Even after this work there remains some uncertainty as to the relative effectiveness of O3 and OH initiation in producing these low-volatility organics from terpenes. This uncertainty seems inherent in the difficulty in absolutely calibrating the CIMS analysis for the various types of species. Resolving that uncertainty will probably require other analytical methods; however, the fact that among the four ionizing species the one clear outlier was the (~40 times) smaller sensitivity of nitrate ionization towards the OH-initiated RO2 products, it seems very unlikely that OH-initiation is at less than rough parity with the O3-initiated processes.

A mechanism for the oxidation is inferred based in part on D exchange reactions aimed at counting the acidic hydrogens in the products, and in part on the current knowledge of the relevant chemical pathways. The picture in scheme I is perhaps a bit speculative, but I think accurately presents the best estimate of this oxidation mechanism. The fact that the experimental results in this paper are consistent with that mechanism is a highly useful piece of information.

There are some small typographical problems, like "predominate" instead of "predominant" on the third page and "Was ist hier falsch?" in reference 17. I recommend a careful edit.

On the whole this is a very thorough set of measurements on an important oxidation system, and the paper will have a high impact.

Reviewer #2 (Remarks to the Author):

Review on "OH radical induced formation of highly oxidized organic Compounds" by Berndt et al.:

This manuscript presents the detection of highly oxidized multifunctional organic compounds (HOMs) due to OH oxidation. The bulk of the results presented focuses on HOMs originating from α-pinene, which can be considered the most important model compound in SOA production experiments. The detection of these HOMs broaden the horizon in terms of possible and realistic oxidation products on a mechanistic level. The production rate detected will improve our understanding of ambient SOA production significantly. There has been a mismatch of SOA production measured in laboratory experiments, SOA concentrations measured in field campaigns, and SOA representation in modelling studies. Multiple studies have shown that extrapolation from laboratory studies significantly underestimate atmospheric SOA production. This undisclosed source of SOA precursors has been a critical issue for long time.

Experiments have been performed in a free-jet flow system and a chemical ionization - atmospheric pressure interface - time-of-flight (CI-APi-ToF) has been used for chemical analysis. The results of this study are based on accurate laboratory experiments and have been assessed against atmospheric measurements. The measurement results are further supported by

computational analysis. While Ehn et al., 2013 could reveal the formation of HOMs from the ozone oxidation of α-pinene, experimental evidence of the production of these compounds due to the oxidation of the most important atmospheric oxidant was still lacking. It should be noted, that a strong indication for an amplification of detectable HOMs by utilizing acetate as reagent ion has been presented by Berndt et al., 2015. Nevertheless, the here presented experimental evidence is of high importance for the atmospheric science community.

This study illustrates the eminent need for thorough/meticulous experimental work and the continuous development and refinement of instrumentation and measurement procedures. I recommend this manuscript for publication after the following comments have been addressed:

General comment:
Though the authors present ambient measurements supporting their laboratory findings it would be appreciated if the authors assess their results on previously published data. There are a couple of ambient studies indicating the big discrepancy between atmospheric measurements and laboratory extrapolations. The following two publications shall be considered exemplary:

1) Volkamer et al., GRL 2006 doi:10.1029/2006GL026899: These authors present discrepancies of up to two orders of magnitude between measured and modeled SOA concentration specifically referring to anthropogenic VOCs. A discussion on the possible impact in the case of transferring the reaction scheme to anthropogenic VOCs would be very interesting.
2) Russel et al., JGR 2007 doi:10.1029/2006JD007736: These authors present a deviation from laboratory extrapolations to ambient measurements of up to two orders of magnitude in the case of most probably α- and β-pinene photooxidation. The authors could illuminate if the new findings could explain this deviation and could contribute to closing the gap.

Specific comments:
Page 1 paragraph 1 line 1: the authors might consider changing "challenging" to "intriguing"
Page 2 paragraph 1 line 6: "both fundamental and quantitative knowledge,..., is → are still missing"
Page 2 paragraph2 line 3: "HOMs can partly be classified..." What does partly mean? In majority? And what is/are the other part/s?
Page 2 paragraph 3 line 2: Depending on the definition of "aerosol" the authors consider rephrasing "SOA constituents" to "SOA precursors" as the APi-TOF investigates the gas phase.
Page 2 paragraph 3 line 5: The authors might elucidate the expression "total signal measurements" (possibly in the supplement) for readers not experts in their measurement technique.
Page 2 paragraph 4 line 12 and 16: The authors might review the listed reference, as it seems they are referring to reference 15 not 14
Page 4 paragraph 5: It would be helpful if the authors guide the reader by mentioning the markers of interest within the description of the figure.
Figure S11: In view of the values indicated by the y-axis the reader might assume that the presented measurement values have already been multiplied by a factor of 40 similar to the presentation in Figure 3 of the manuscript. If this is the case, it should be stated clearly. If this is not the case, a short discussion of the high ambient concentration should be added.

Reviewer #3 (Remarks to the Author):

This paper describes new measurements of highly functionalized atmospheric organic radical species, formed in the OH-initiated oxidation of monoterpenes. Chemical ionization mass spectrometry with a new ionization scheme (utilizing the conjugate bases of organic acids) is employed to demonstrate the formation of these compounds in both laboratory studies and the field. It is found that the yields of such functionalized species were higher than previously thought

(since they were not detected efficiently by other techniques), and thus may play a role in aerosol formation and growth. This is not a terribly surprising result - previous work (ref 6) showed that such multifunctional species were formed from O3+terpenes, so similar products from OH+terpenes are completely expected - but it is a true improvement over previous work. However, I have some concerns about the results, particularly the apparent long lifetime of the key peroxy radical examined in this work, and an apparent major difference between the field and laboratory results. These need to be addressed if this manuscript is to be published in Nature Communications.

1) An important feature of the HO-C10H15(OO)(OOH)O2 radical (henceforth "RO2"), which is not even mentioned in the manuscript, is its apparent atmospheric persistence. From Figure S1, its lifetime clearly exceeds 8s. It apparently is much longer than this, since the mass spectra after 8s (Fig 2) show substantial RO2 but very little (if any) closed-shell HOM products. In fact, the slow decays in the RO2 signal in the ambient (Fig 3a, over the course of each afternoon/evening/night) suggest lifetimes on the order of several hours! This strikes me as highly unusual, and needs to be addressed in this manuscript. Key questions include:

i) Why is this one RO2 species so long-lived? It seems to me that additional isomerization reactions could easily occur. Quantum chemical calculations are needed to show that these will not occur on short (second-to-minute) timescales.

ii) Conversely, why are the precursor peroxy species (in Fig 1b) apparently so short-lived? Based on the rapid formation of the RO2, these precursors must have unimolecular lifetimes of no more than 1s. Again, quantum chemical calculations could help.

iii) In both the field and the laboratory, what are the approximate lifetimes of these species against bimolecular channels (RO2+NO, RO2+HO2, RO2+RO2)? I understand there is some uncertainty as to the concentrations of these co-reactants (and rate constants), but it still needs to be shown that its reasonable to expect such reactions to be negligibly slow on timescales of minutes to hours.

- Figure S9 shows that the RO2 concentration drops by about an e-fold when 2e10 molec/cm3 of NO is present (presumably due to RO2+NO, reactions 8-9). This corresponds to a RO2+NO rate constant of ~7e-12 cm3/molec/s, which is reasonably consistent with other organic peroxy+NO rate constants. However, no such dependence is seen in the field (Figure 3). The day of 29/03 starts with [NO] up at 2e10 molec/cm3, which then suddenly drops to ~0 at midday. At that point (since OH is still present) there should be a large spike in [RO2] (by a factor of ~3) - but no such change is observed (it actually drops by a factor of 2). Thus the laboratory and field studies appear to be telling very different stories about RO2 reactivity, and thus the two studies may be looking at different species, and/or different chemical pathways. This discrepancy, and its implications, needs to be addressed and discussed in detail.

Other comments

Fig S1: the fits seem to be forced through zero. An unconstrained linear fit would seem to indicate a slightly positive intercept (i.e., some ion signal with no reaction). What could be causing this?

Figure 2: The y axes of these plots are confusing. Naively, the plots would seem to indicate that [RO2] in the flow tube is dependent on the ionization technique used. Obviously this is not correct - the difference is in the inferred concentration from the different ionization schemes, which each use the calibration approach outlined in the SI. The axis label and figure caption should be changed to make this clear. (Maybe a term such as "sulfuric-acid-equivalent" concentration would help?)

Scheme 1b: the very last reaction seems to me to be unlikely, as the bridgehead hydrogen does not seem to be easily accessible to the peroxy radical center. Have quantum chemical calculations been done to show this pathway is indeed possible?

Second-to-last page, middle (and Table S2): two of these compounds are actually of reasonably high volatilities (vapor pressures on the order of ppb), so are best described as semivolatile compounds.

Then it is stated that these oxidation products "can explain a substantial fraction of SOA formation from low-NO a-pinene photooxidation experiments". It's unclear to me how this statement follows from the results, or from the two cited papers. To the best of my knowledge, these compounds have not been detected in the SOA. Further, elemental analysis of such SOA (e.g., Chhabra et al, ACP 11:8827, 2011) finds O:C ratios that are substantially lower than those of the HOM molecules described. So I do not see the clear connection between this study and other previous SOA work.

Second-to-last-page, bottom: I disagree with the sentence that the RO2 levels "followed strictly the global radiation" at the TROPOS station (Figure S11). Yes, there's a strong diurnal trend, but there are day-to-day differences in [RO2] that are in no way related to differences in radiation. This could be from any number of confounding factors (emissions, transport, [NO], etc.); these should be mentioned and the wording changed.

SI, Eq. 1: none of the terms (most importantly f_HOM) are defined. The other terms are in brackets, which implies concentrations - I assume "ion signal" is meant here instead?

Fig 3: for context, [O3] needs to be shown here as well. For example, the peroxy radicals from ozonolysis spike during the high-NO period (mentioned above), which is counterintuitive.

Throughout, the language of the paper is rather awkward, and at times hard to follow. (For example see the last sentence of the abstract, the first sentence of the intro, and the last sentence of the paper.) Additional editing for language and clarity would be helpful.

Reviewers' comments to author:

Reviewer: 1

Comments:
This manuscript uses a combination of laboratory and field measurements to demonstrate that OH-initiated oxidation is a substantial source of low-volatility organic compounds and that this pathway must be considered in models of organic aerosol formation in the troposphere. The key insight is that the ion used in chemical ionization mass spectrometry can show marked differences in sensitivity to differently functionalized species, and the authors therefore introduce a suite of ionizing methods to analyze the oxidation process, and compare pure OH-initiation with the combined OH and O3 initiation inherent in ozonolysis experiments. This is a very thorough set of research, and despite the complexity of the oxidation systems the important conclusions about the importance of OH-initiation and about the likely mechanism of oxidation are in my view robust.

Let's look at these conclusions in turn. Even after this work there remains some uncertainty as to the relative effectiveness of O3 and OH initiation in producing these low-volatility organics from terpenes. This uncertainty seems inherent in the difficulty in absolutely calibrating the CIMS analysis for the various types of species. Resolving that uncertainty will probably require other analytical methods; however, the fact that among the four ionizing species the one clear outlier was the (~40 times) smaller sensitivity of nitrate ionization towards the OH-initiated RO2 products, it seems very unlikely that OH-initiation is at less than rough parity with the O3-initiated processes.

A mechanism for the oxidation is inferred based in part on D exchange reactions aimed at counting the acidic hydrogens in the products, and in part on the current knowledge of the relevant chemical pathways. The picture in scheme I is perhaps a bit speculative, but I think accurately presents the best estimate of this oxidation mechanism. The fact that the experimental results in this paper are consistent with that mechanism is a highly useful piece of information.

There are some small typographical problems, like "predominate" instead of "predominant" on the third page and "Was ist hier falsch?" in reference 17. I recommend a careful edit.

On the whole this is a very thorough set of measurements on an important oxidation system, and the paper will have a high impact.

<u>Reply:</u> The reviewer is right (in line with the other reviewers), that the reaction scheme is probably a bit speculative. So, scheme 1 was revised and extended based on latest results from quantum chemical calculations performed in the course of the revision process. The manuscript was carefully edited.

Reviewer: 2

Comments:

This manuscript presents the detection of highly oxidized multifunctional organic compounds (HOMs) due to OH oxidation. The bulk of the results presented focuses on HOMs originating from α-pinene, which can be considered the most important model compound in SOA production experiments. The detection of these HOMs broaden the horizon in terms of possible and realistic oxidation products on a mechanistic level. The production rate detected will improve our understanding of ambient SOA production significantly. There has been a mismatch of SOA production measured in laboratory experiments, SOA concentrations measured in field campaigns, and SOA representation in modelling studies. Multiple studies have shown that extrapolation from laboratory studies significantly underestimate atmospheric SOA production. This undisclosed source of SOA precursors has been a critical issue for long time.

Experiments have been performed in a free-jet flow system and a chemical ionization - atmospheric pressure interface - time-of-flight (CI-APi-ToF) has been used for chemical analysis. The results of this study are based on accurate laboratory experiments and have been assessed against atmospheric measurements. The measurement results are further supported by computational analysis. While Ehn et al., 2013 could reveal the formation of HOMs from the ozone oxidation of α-pinene, experimental evidence of the production of these compounds due to the oxidation of the most important atmospheric oxidant was still lacking. It should be noted, that a strong indication for an amplification of detectable HOMs by utilizing acetate as reagent ion has been presented by Berndt et al., 2015. Nevertheless, the here presented experimental evidence is of high importance for the atmospheric science community.

This study illustrates the eminent need for thorough/meticulous experimental work and the continuous development and refinement of instrumentation and measurement procedures. I recommend this manuscript for publication after the following comments have been addressed:

General comment:

Though the authors present ambient measurements supporting their laboratory findings it would be appreciated if the authors assess their results on previously published data. There are a couple of ambient studies indicating the big discrepancy between atmospheric measurements and laboratory extrapolations. The following two publications shall be considered exemplary:

1) Volkamer et al., GRL 2006 doi:10.1029/2006GL026899: These authors present discrepancies of up to two orders of magnitude between measured and modeled SOA concentration specifically referring to anthropogenic VOCs. A discussion on the possible impact in the case of transferring the reaction scheme to anthropogenic VOCs would be very interesting.
2) Russel et al., JGR 2007 doi:10.1029/2006JD007736: These authors present a deviation from laboratory extrapolations to ambient measurements of up to two orders of magnitude in the case of most probably α- and β-pinene photooxidation. The authors could illuminate if the new findings could explain this deviation and could contribute to closing the gap.

Reply: Yes, we agree that the presence of high HOM concentrations from the OH-initiated oxidation of BVOCs can help to explain parts of the missing SOA formation in the atmosphere. But, it is to be noted, that our findings cannot be transferred to other reaction systems. We added a discussion on that to the Discussion paragraph referring to the publications by Russel et al., 2007 and Volkamer et al., 2006.

Changes in the text: Furthermore, several studies indicate a strong quantitative disagreement between experimentally observed SOA formation from field campaigns and model simulations based on laboratory data. For instance, Russel *et al.*[32] observed for photooxidation conditions, most likely dominated by the OH radical reactions of α- and β-pinene, up to two orders of magnitude higher SOA generation compared to model predictions. The results of our study are able to overcome this discrepancy, at least qualitatively. But also for urban areas

with prevalent anthropogenic emissions, much larger amounts of SOA are reported than predicted by models.[33] Volkamer *et al.*[33] determined that a significant fraction of the excess SOA was formed from first-generation oxidation products of the anthropogenic emissions. It could be speculated that similar reaction pathways of HOM formation, as shown in our study for OH + α-/β-pinene, take place in the course of the degradation of anthropogenic emissions, such as aromatics. However, a simple transfer of our results to other reaction systems is impossible due to the substance-specific reactivity of the RO_2 isomerization steps. Especially in this field, much more experimental and theoretical work is needed in order to clarify the formation pathways of SOA precursors generated from anthropogenic emissions.

Specific comments:

Page 1 paragraph 1 line 1: the authors might consider changing "challenging" to "intriguing"

Reply: We changed the wording accordingly.

Page 2 paragraph 1 line 6: "both fundamental and quantitative knowledge,..., is → are still missing"

Reply: We changed the typo accordingly

Page 2 paragraph2 line 3: "HOMs can partly be classified..." What does partly mean? In majority? And what is/are the other part/s?

Reply: The other parts are less volatile organic compounds such as LVOCs (low volatile organic compounds) or SVOCs (semi-volatile organic compounds). The fraction of HOMs belonging to ELVOCs cannot be quantified because accurate vapor pressures are not known.

Changes in the text: The remaining fraction can be attributed to low- or semi-volatile organic compounds. An exact determination of the vapour pressure of HOMs is currently impossible preventing a more accurate classification.

Page 2 paragraph 3 line 2: Depending on the definition of "aerosol" the authors consider rephrasing "SOA constituents" to "SOA precursors" as the APi-TOF investigates the gas phase.

Reply: We agree with the reviewer and changed the wording.

Page 2 paragraph 3 line 5: The authors might elucidate the expression "total signal measurements" (possibly in the supplement) for readers not experts in their measurement technique.

Reply: A short explanation for "total signal measurement" was added.

Changes in the text: Estimated molar HOM yields of less than 1%[5] and $0.44^{+0.44}_{-0.22}$ %[10] from OH + α-pinene are reported using total signal measurements, i.e. all appearing signals in a selected mass-to-charge range were taken into account without a specification of the respective reaction product.

Page 2 paragraph 4 line 12 and 16: The authors might review the listed reference, as it seems they are referring to reference 15 not 14

Reply: The reviewer is right that we referred to reference 15 not to 14. Thanks for attentive reading! The authors carefully checked the reference list again.

Page 4 paragraph 5: It would be helpful if the authors guide the reader by mentioning the markers of interest within the description of the figure.

Reply: Now the colour coding for different species concentrations is also mentioned in the text.

Figure S11: In view of the values indicated by the y-axis the reader might assume that the presented measurement values have already been multiplied by a factor of 40 similar to the presentation in Figure 3 of the manuscript. If this is the case, it should be stated clearly. If this is not the case, a short discussion of the high ambient concentration should be added.

Reply: The reviewer is right that the stated HOM-RO_2 concentration in Melpitz is much higher than the concentration found in Hyytiälä. The given concentrations in the new Figure S12 (former Figure S 11) are the original measurement data using nitrate ionization without a correction by a factor of 40. The relatively large difference can be due to a couple of reasons: First, Melpitz is a rural background station whereas Hyytiälä is located in a remote area and the global radiation, connected to the OH radical concentration, in July in Melpitz was much higher than during the Hyytiälä measurements in March. Secondly, it is expected that the monoterpene emission, and consequently the resulting monoterpene concentrations, in the beginning of spring in Hyytiälä were definitely smaller than those in Melpitz in the summertime. That means that both reactant concentrations needed to form the HOM-RO_2 radicals were most likely higher in Melpitz. Unfortunately, a more quantitative explanation is impossible due to the lack of measurement data.

Changes in SI: The given concentrations are the original measurement data using nitrate ionization without a correction by a factor of 40. The relatively large difference of the HO-$C_{10}H_{15}(OO)(OOH)O_2$ radical concentrations comparing Melpitz with Hyytiälä data (Fig. 3a) can be due to a couple of reasons: First, Melpitz is a rural background station whereas Hyytiälä is located in a remote area and the global radiation, connected to the OH radical concentration, in July in Melpitz was much higher than during the Hyytiälä measurements in March. Secondly, it is expected that the monoterpene emission, and consequently the resulting monoterpene concentrations, in the beginning of spring in Hyytiälä were definitely smaller than those in Melpitz in the summertime. That means that both reactant concentrations needed to form the HOM-RO_2 radicals were most likely higher in Melpitz. A more quantitative explanation including an assessment of the loss processes is impossible due to the lack of measurement data.

Reviewer: 3

Comments:

This paper describes new measurements of highly functionalized atmospheric organic radical species, formed in the OH-initiated oxidation of monoterpenes. Chemical ionization mass spectrometry with a new ionization scheme (utilizing the conjugate bases of organic acids) is employed to demonstrate the formation of these compounds in both laboratory studies and the field. It is found that the yields of such functionalized species were higher than previously thought (since they were not detected efficiently by other techniques), and thus may play a role in aerosol formation and growth. This is not a terribly surprising result - previous work (ref 6) showed that such multifunctional species were formed from O3+terpenes, so similar products from OH+terpenes are completely expected - but it is a true improvement over previous work. However, I have some concerns about the results, particularly the apparent long lifetime of the key peroxy radical examined in this work, and an apparent major difference between the field and laboratory results. These need to be addressed if this manuscript is to be published in Nature Communications.

1) An important feature of the HO-C10H15(OO)(OOH)O2 radical (henceforth "RO2"), which is not even mentioned in the manuscript, is its apparent atmospheric persistence. From Figure S1, its lifetime clearly exceeds 8s. It apparently is much longer than this, since the mass spectra after 8s (Fig 2) show substantial RO2 but very little (if any) closed-shell HOM products. In fact, the slow decays in the RO2 signal in the ambient (Fig 3a, over the course of each afternoon/evening/night) suggest lifetimes on the order of several hours! This strikes me as highly unusual, and needs to be addressed in this manuscript. Key questions include:

Reply: Yes, the lifetime of the HO-$C_{10}H_{15}$(OO)(OOH)O_2 radicals with respect to further RO_2 isomerization steps is larger than 8 s as experimentally determined from the free-jet investigations. This fact is not so surprising because the isomerization rate coefficients can span a wide range strongly dependent on the chemical structure of the RO_2 radicals. Theoretical calculations for a couple of sample RO_2 radicals are given by Crounse et al., ref. (21) in this manuscript. From ambient measurement it is almost impossible to conclude any RO_2 lifetimes. The atmosphere is not a well-defined laboratory experiment! In the atmosphere we do not know exactly the source (e.g. [OH]) nor the sink terms (e.g. [RO_2], [HO_2]) and their variability. And also air mass changes need to be considered (see below).

Changes in the text: The variability of the conditions in the atmosphere, i.e. changing air masses with changing trace gas composition, and the lack of precise terpene and OH radical concentrations do not allow a more accurate analysis of the field data.

i) Why is this one RO2 species so long-lived? It seems to me that additional isomerization reactions could easily occur. Quantum chemical calculations are needed to show that these will not occur on short (second-to-minute) timescales.

Reply: As said in the answering before, the rate of the isomerization steps is strongly dependent on the RO_2 structure. And obviously, for these HO-$C_{10}H_{15}$(OO)(OOH)O_2 radicals the lifetime exceeds few 10 seconds or more. Comprehensive theoretical calculations regarding further reaction of the seven oxygen containing RO_2 radicals are given now in the Supplementary Information.

Changes in SI: Paragraph 3 in Supplementary Information.

ii) Conversely, why are the precursor peroxy species (in Fig 1b) apparently so short-lived? Based on the rapid formation of the RO2, these precursors must have unimolecular lifetimes of no more than 1s. Again, quantum chemical calculations could help.

Reply: Yes, the precursor RO_2 of the detected HO-$C_{10}H_{15}(OO)(OOH)O_2$ radicals have to have unimolecular lifetimes in the order of 1 s or less. Otherwise we could not measure a linear increase of the HO-$C_{10}H_{15}(OO)(OOH)O_2$ signal with time in the range of 3.0 - 7.9 s, see Figure S1. And also here, theoretical calculations have been conducted supporting the experimental findings, see Paragraph 3 in Supplementary Information.

Changes in the text: Possible reaction pathways for the generation of the proposed highly oxidized RO_2 radical from OH+α-pinene, are shown in Supplementary Schemes S1 - S4. The pathways include an initial intramolecular H-shift,[5,9,15,16,21] followed by O_2 addition leading to RO_2 radicals with five oxygen atoms. Then a very rapid H-shift involving the OOH group[22] takes place followed by an endo-cyclization and a next O_2 addition to get the RO_2 radicals 19, 20 and 29 containing seven oxygene each. Based on quantum chemical calculations (see Supplementary Information), the RO_2 radicals 19, 20 or 29, all with the chemical composition HO-$C_{10}H_{15}(OO)(OOH)O_2$, can be formed at a seconds time scale starting from the RO_2 radical 4 as illustrated in a simplified way in Scheme 1b. The calculations furthermore indicate that 19, 20 and 29 are relatively stable with respect to further isomerization steps (H-shifts).
and
Paragraph 3 in Supplementary Information.

iii) In both the field and the laboratory, what are the approximate lifetimes of these species against bimolecular channels (RO2+NO, RO2+HO2, RO2+RO2)? I understand there is some uncertainty as to the concentrations of these co-reactants (and rate constants), but it still needs to be shown that its reasonable to expect such reactions to be negligibly slow on timescales of minutes to hours.

Reply: Generally, the idea of the experiment is to conduct the reaction under condition of negligible bimolecular RO_2 reactions. That´s clearly stated in the manuscript and the good linearity of RO_2 vs. converted terpene in Figure 2 confirms that. So, from the laboratory experiments we cannot draw any conclusions regarding the RO_2 lifetimes with respect to bimolecular loss processes. And from the measured field RO_2 data we cannot extract any information, as already mentioned in the fist reply to reviewer 3. Here, only very rough estimates are possible assuming any steady state concentrations for NO, HO_2 and total-RO_2 and commonly used values for the corresponding rate coefficients. Doing that, the resulting lifetimes are in the order of minutes.

- Figure S9 shows that the RO2 concentration drops by about an e-fold when 2e10 molec/cm3 of NO is present (presumably due to RO2+NO, reactions 8-9). This corresponds to a RO2+NO rate constant of ~7e-12 cm3/molec/s, which is reasonably consistent with other organic peroxy+NO rate constants. However, no such dependence is seen in the field (Figure 3). The day of 29/03 starts with [NO] up at 2e10 molec/cm3, which then suddenly drops to ~0 at midday. At that point (since OH is still present) there should be a large spike in [RO2] (by a factor of ~3) - but no such change is observed (it actually drops by a factor of 2). Thus the laboratory and field studies appear to be telling very different stories about RO2 reactivity, and thus the two studies may be looking at different species, and/or different chemical pathways. This discrepancy, and its implications, needs to be addressed and discussed in detail.

Reply: Yes, from the well-defined laboratory experiments with addition of the second reactant for the bimolecular reaction of RO_2 with NO (and keeping all other parameters constant!) we are able to figure out information on the RO_2 reactivity with respect to this reaction. But once again, from the field measurements we cannot conclude that with decreasing NO the RO_2 signal should increase simultaneously. Here we have no control of all other reaction parameters. Moreover, especially at the day 29.3. we have to expect that with the rapid drop of the high NO concentration (NO should come from very polluted air masses!) also a change of the air masses took place. And an air mass change, connected with a variation of all trace gas concentrations, results most likely in a significant change of the source and sink terms for the RO_2 radicals. So, we cannot agree with the reviewer´s conclusion that the lab and field data "telling very different stories". From our perspective, the stated

field measurements in Hyttiälä and Melpitz showed very nicely the occurrence of the expected signal of the HO-$C_{10}H_{15}(OO)(OOH)O_2$ radicals and the expected trends as well. Further conclusions seem to be impossible due to the variability in the reaction system.

Other comments

Fig S1: the fits seem to be forced through zero. An unconstrained linear fit would seem to indicate a slightly positive intercept (i.e., some ion signal with no reaction). What could be causing this?

Reply: Linear regression analysis yields (free parameter for the intercept) almost identical lines compared with those shown in the plot (forced through the origin). For instance, for the HO-$C_{10}H_{15}(OO)(OOH)O_2$ radical, the intercept is small and not significant, being $(1.5 \pm 3.0) \times 10^5$ molecules cm^{-3}. While the RO_2 detection seems to be very sensitive (with a detection limit down to 10^4 molecules cm^{-3}!) with a good reproducibility, the measurements are not totally perfect!

Figure 2: The y axes of these plots are confusing. Naively, the plots would seem to indicate that [RO2] in the flow tube is dependent on the ionization technique used. Obviously this is not correct - the difference is in the inferred concentration from the different ionization schemes, which each use the calibration approach outlined in the SI. The axis label and figure caption should be changed to make this clear. (Maybe a term such as "sulfuric-acid-equivalent" concentration would help?)

Reply: Yes we know, the calibration factor, and subsequently the value of the resulting concentration, is a critical issue. Absolute calibration is impossible. The calculated lower end value of the calibration factor (that is in line with the absolute calibration for H_2SO_4 in our system) is stated in the Method section. The resulting concentrations are called "estimated concentrations" at different places in the text and SI. The figure caption of Figure 2 was changed accordingly.

Changes in thr text: Figure 2: Estimated concentrations of highly oxidized RO_2 radicals as a function of reacted a) α-pinene and b) β-pinene. The given concentrations for the different reagent ions were obtained using a calibration factor from absolute sulfuric acid calibration that is in line with the calculated, lower end calibration factor of an ion-molecule reaction. …
and
The stated radical concentrations, and consequently the yields, represent estimated, lower end values for the different ionization schemes.

Scheme 1b: the very last reaction seems to me to be unlikely, as the bridgehead hydrogen does not seem to be easily accessible to the peroxy radical center. Have quantum chemical calculations been done to show this pathway is indeed possible?

Reply: In the course of the manuscript revision, comprehensive quantum chemical calculations have been performed qualifying possible reaction pathways for the HO-$C_{10}H_{15}(OO)(OOH)O_2$ radical formation and its relatively long lifetime much better. Needed changes have been done in the text.

Changes in the text: Possible reaction pathways for the generation of the proposed highly oxidized RO_2 radical from OH+α-pinene, are shown in Supplementary Schemes S1 - S4. The pathways include an initial intramolecular H-shift,[5,9,15,16,21] followed by O_2 addition leading to RO_2 radicals with five oxygen atoms. Then a very rapid H-shift involving the OOH group[22] takes place followed by an endo-cyclization and a next O_2 addition to get the RO_2 radicals 19, 20 and 29 containing seven oxygene each. Based on quantum chemical calculations (see Supplementary Information), the RO_2 radicals 19, 20 or 29, all with the chemical composition HO-$C_{10}H_{15}(OO)(OOH)O_2$, can be formed at a seconds time scale starting from the RO_2 radical 4 as illustrated in a simplified way in Scheme 1b. The calculations furthermore indicate that 19, 20 and 29 are relatively stable with respect to further isomerization steps (H-shifts).

and
Paragraph 3 in Supplementary Information.

Second-to-last page, middle (and Table S2): two of these compounds are actually of reasonably high volatilities (vapor pressures on the order of ppb), so are best described as semivolatile compounds.

Reply: The calculated vapour pressures of the proposed reaction products are now below 10^{-9} atm at 295 K corresponding to a mixing ratio of < 1 ppb. Together with accretion products (now added in the revised text), the vapour pressures range from 8×10^{-10} to 2×10^{-18} atm. These detected reaction products all together can be regarded as "low-volatile" substances in a general term. We did not intend to link the vapour pressures to the volatility scheme given e.g. by Pandis et al. Faraday Discuss., 2013, 165, 9. In this scheme, the vapour pressures of the HOM compounds and accretion products range from semi- to extremely low-volatility compounds. However, we only want to state that these HOMs have a low volatility. We changed the sentence to the following:

Changes in the text: Thus, these highly oxidized products have a low volatility (and are water soluble), and can effectively condense on surfaces.

Then it is stated that these oxidation products "can explain a substantial fraction of SOA formation from low-NO a-pinene photooxidation experiments". It's unclear to me how this statement follows from the results, or from the two cited papers. To the best of my knowledge, these compounds have not been detected in the SOA. Further, elemental analysis of such SOA (e.g., Chhabra et al, ACP 11:8827, 2011) finds O:C ratios that are substantially lower than those of the HOM molecules described. So I do not see the clear connection between this study and other previous SOA work.

Reply: It was said "can explain …". The referee is right, that we have no experimental evidence of that at the moment. But it is very likely, that the detected HOMs participate in the process of SOA formation. The HOMs (containing OOH groups!) are expected to be processed in the particle phase forming more stable substances. It is unlikely, that hydroperoxides are stable enough to survive for a longer time. Thus, O:C ratios of particles, as measured e.g. by Chhabra et al. by means of an AMS, do not reflect the O:C ratio of the gas-phase species taking part in particle growth. These O:C ratios represent an average number for all components in the particle phase after any processing. Regarding the cited papers: The Caltech experiments for low-NO conditions, now ref.28, showed SOA formation independent of OH exposure and the "traditional modelling" failed explaining the experimental findings. So, the authors took into consideration also other SOA formation routes, like autoxidation.

Changes in the text: Recently, SOA yields of 17 - 26 % from low-NO α-pinene photooxidation experiments in the Caltech chamber were reported being independent of OH exposure.[28] This finding was not in line with SOA modelling results for this reaction system indicating the presence of other SOA formation routes, like an autoxidation mechanism,[5,9,21] not implemented in modelling schemes yet.[28] Our lower limit molar HOM yield from OH + α-pinene of 2.4 % corresponds to a lower limit mass yield of 4.4 % (average HOM mass: 250 g mol^{-1}). This mass yield can explain at least a fraction of the measured SOA yield from the Caltech experiments[28] and also from other SOA studies[29].

Second-to-last-page, bottom: I disagree with the sentence that the RO2 levels "followed strictly the global radiation" at the TROPOS station (Figure S11). Yes, there's a strong diurnal trend, but there are day-to-day differences in [RO2] that are in no way related to differences in radiation. This could be from any number of confounding factors (emissions, transport, [NO], etc.); these should be mentioned and the wording changed.

Reply: Yes, "followed strictly" is a bit too strong. The wording was changed accordingly.

Changes in the text: Here, the diurnal pattern of the HO-$C_{10}H_{15}(OO)(OOH)O_2$ radical followed the diurnal trend of the global radiation which was again used as a proxy for OH (Supplementary Fig. S12).
and

The variability of the conditions in the atmosphere, i.e. changing air masses with changing trace gas composition, and the lack of precise terpene and OH radical concentrations do not allow a more accurate analysis of the field data.

SI, Eq. 1: none of the terms (most importantly f_HOM) are defined. The other terms are in brackets, which implies concentrations - I assume "ion signal" is meant here instead?

Reply: A definition of the calculated calibration factor $f_{HOM,calc}$ and for the term in brackets is given in the revised text.

Changes in the text: The lower end value of the calibration factor f_{HOM} can be calculated considering the $(HOM)X^-$ adduct formation in the CI-inlet via reaction (1), $f_{HOM,calc} = 1 / (k \times t \times f_{inlet})$[5,41], where k is the rate coefficient of the ion-molecule reaction, t the reaction time and the term f_{inlet} considers the sample (HOM) loss in the sampling tube.

$$HOM + (HX)_nX^- \rightarrow (HOM)X^- + (HX)_n \quad, n = 0,1,2 \qquad (1)$$

The rate coefficient $k = k_1$ is set to $k_1 = (2 - 3) \times 10^{-9}$ cm^3 $molecule^{-1}$ s^{-1}, typical for a series of ion-molecule reactions close to the collision limit[42,43]. Taking into account a 12% diffusion loss of the sample (HOM) in the short sampling tube (diffusion controlled wall loss for an assumed diffusion coefficient $D = 0.08$ cm^2 s^{-1}), $f_{inlet} = 0.88$, and a reaction time of the ion-molecule reaction t = 0.2 - 0.3 s, $f_{HOM,calc} = (1.3 - 2.8) \times 10^9$ molecules cm^{-3} follows.
and
The values given in the brackets are the measured ion signals.

Fig 3: for context, [O3] needs to be shown here as well. For example, the peroxy radicals from ozonolysis spike during the high-NO period (mentioned above), which is counterintuitive.

Reply: The ozone concentration showed only small variation during the measurement campaign in Hyytiälä. The ozone time series is given now in Figure S11 in SI. The high-NO period in the morning hours of 29.3. is most likely connected with an air mass change. NO concentrations of 2×10^{10} molecules cm^{-3} or higher are unusual values in this area. We do not know how this air mass change influenced the concentrations of the RO_2 radicals. Nevertheless, the presence of really high NO concentrations showed the occurrence of the expected organic nitrates. Because of that are the measurements so interesting.

Throughout, the language of the paper is rather awkward, and at times hard to follow. (For example see the last sentence of the abstract, the first sentence of the intro, and the last sentence of the paper.) Additional editing for language and clarity would be helpful.

Reply: The mentioned sentences were removed or reformulated. The whole manuscript was carefully edited.

Reviewers' comments:

Reviewer #2 (Remarks to the Author):

The authors have addressed the reviewers' comments appropriately. I consider their revision of the manuscript and the point by point responds satisfactory and recommend the current manuscript for publication.

Reviewer #3 (Remarks to the Author):

This revised paper is improved, especially with regards to the discussion of the unimolecular kinetics of the different RO2 radicals (with 3O's, 5Os, or 7O's). However my original questions about lifetimes of RO2 radicals versus bimolecular reactions were for the most part not addressed; I describe them below in more detail. I view this as a crucial component of the manuscript – the competition between bimolecular and unimolecular reactions provides important constraints on the kinetics of the unimolecular reactions, and indeed serves as a test of the entire mechanism put forth, since the kinetics of bimolecular reactions (RO2+NO, RO2+HO2, RO2+RO2) are reasonably well-understood.

1) RO2 lifetimes in the laboratory. In my original review I requested estimates of lifetimes of RO2 vs. reaction with NO, HO2, and RO2, to compare against unimolecular rates. This provides insights into, and constraints on, the isomerization kinetics. The authors responded that "the idea of the experiment is to conduct the reaction under condition of negligible bimolecular RO2 reactions"… but then (bottom of p. 4) have an entire section entitled "RO2 + NO reaction"! This section includes information on RO2+NO, RO2+HO2, RO2+RO2 – all the reactions I requested information about. So I don't understand how this section is at all consistent with the authors' assertion that these reactions are negligible. Again, these lifetimes need to be estimated, to show consistency with the inferred isomerization kinetics. This is a powerful, and necessary, test of the mechanism put forth.

2) RO2 lifetimes in the field. I agree that accurate RO2 lifetimes cannot be deduced from field data - and of course the authors are correct that the atmosphere is not a "well-defined laboratory experiment" - but that does not mean that qualitative conclusions cannot be made! The data clearly show that there is an exceedingly slow decay (on the order of hours) of the RO2 radical in question at night. There are only two ways this can be the case: either there is some sort of nighttime source (somewhere in the local/regional atmosphere), or the radical has a lifetime of several hours. If it's the former, there must be a substantial nighttime OH source, or a pathway to RO2 formation by some other channel; if it's the latter, then these RO2 radicals are so slow to isomerizes that bimolecular reactions with almost certainly win out. Which is it? Regardless, this is highly unusual behavior for OH-derived radicals in the nighttime, and so cannot simply be left unmentioned in the text.

Also, the authors mention in the rebuttal that the bimolecular lifetimes are on the order of minutes. Because of the importance of bimolecular lifetimes to this whole story, this needs to go in the main text as well.

Other points

P5, 1st full para: I don't see how autoxidation can explain the McVay et al. (ref. 28) results. Their RO2+HO2 lifetimes were 10s for the high-UV case, 50s for the low-UV case. In the present study the unimolecular lifetime of the RO2 radical is thought to be 10s or longer. It would have to be considerably shorter than that to compete with HO2 reaction, such that yield was independent of UV intensity.

P6, 1st full para: The choice of these two references (32 and 33) to discuss the underprediction of SOA by models seems highly unusual. There is a large body of literature describing such underpredictions, and neither of these articles were the first. The first such article was Heald et al. 2005, GRL 32:L18809 2005. (Also, "Russell" is misspelled.)

P7, 2nd para: This discussion is highly repetitive of what is stated previously (top of p. 4). Moreover, it is quite misleading. Refs. 34 and 35 say little to nothing about sequential RO2 isomerizations, but instead focus on the initial isomerization (RO2->QOOH) only. (I don't have access to ref 23.) In combustion, the resulting OOQOOH radical typically doesn't undergo additional isomerization reactions, but rather decomposes, giving OH and other products. (This is actually made very clear in ref 35!) Also, while the combustion community has known about ROO->QOOH reactions for a long time, but vast majority of that work (until recently) was for R=alkyl groups. In that case, the C-H bond is sufficiently strong that isomerization won't happen at 298K – this is why atmospheric chemists, also focused initially on alkane oxidation, did not consider it. Only as the focus has shifted to the oxidation reactions of oxygenates (with weaker C-H bonds) has the importance of such isomerization reactions become clear.

Reviewer #3 (Remarks to the Author):

This revised paper is improved, especially with regards to the discussion of the unimolecular kinetics of the different RO2 radicals (with 3O's, 5Os, or 7O's). However my original questions about lifetimes of RO2 radicals versus bimolecular reactions were for the most part not addressed; I describe them below in more detail. I view this as a crucial component of the manuscript – the competition between bimolecular and unimolecular reactions provides important constraints on the kinetics of the unimolecular reactions, and indeed serves as a test of the entire mechanism put forth, since the kinetics of bimolecular reactions (RO2+NO, RO2+HO2, RO2+RO2) are reasonably well-understood.

1) RO2 lifetimes in the laboratory. In my original review I requested estimates of lifetimes of RO2 vs. reaction with NO, HO2, and RO2, to compare against unimolecular rates. This provides insights into, and constraints on, the isomerization kinetics. The authors responded that "the idea of the experiment is to conduct the reaction under condition of negligible bimolecular RO2 reactions"… but then (bottom of p. 4) have an entire section entitled "RO2 + NO reaction"! This section includes information on RO2+NO, RO2+HO2, RO2+RO2 – all the reactions I requested information about. So I don't understand how this section is at all consistent with the authors' assertion that these reactions are negligible. Again, these lifetimes need to be estimated, to show consistency with the inferred isomerization kinetics. This is a powerful, and necessary, test of the mechanism put forth.

Reply: We are grateful for the provided clarifications, as we now better understand the concerns of the reviewer on this point, and are able to provide an improved response. We realize that it was not made clear enough how to distinguish between the different sets of experiments in the previous manuscript versions, and we have tried to amend this in the revised version.
The basic idea of this work was to conduct free-jet experiments with OH radical reactions under conditions of negligible bimolecular RO_2 reactions. And these unwanted bimolecular RO_2 steps were indeed absent in the sets of experiments using ozonolysis-based OH radical formation and most likely also in the "soft" H_2O_2 photolysis runs made in the free-jet apparatus. The absence of these unwanted RO_2 reactions is clearly seen i) in the linear RO_2 rise with time in fig.S1, ii) in the linear RO_2 rise with increasing terpene conversion in fig.2 and iii) from the absence of RO_2 product signals in the mass spectra recorded under these conditions, i.e. in fig.1, fig.S6 and fig.S7.
A paragraph was added to "Methods" showing the results of a rough estimate of the RO_2 lifetimes with respect to the bimolecular reactions with other RO_2, HO_2 and NO under these reaction conditions. Also these calculations show that bimolecular RO_2 step can be neglected.
Changes in the text:
"**Unwanted bimolecular RO_2 radical reactions in the free-jet experiments**. The basic idea of the ozonolysis-based OH radical experiments performed in the free-jet flow system was to conduct the reaction under conditions of negligible bimolecular RO_2 reactions. We are not able to measure total RO_2 and HO_2 radical concentrations in our experiment. As a

conservative estimate, it is assumed that the total RO_2 as well as the HO_2 radical concentration did not exceed the amount of reacted terpene of $< 8 \times 10^{8}$ molecules cm^{-3}. Rate coefficient of the $RO_2 + RO_2$ and the $HO_2 + RO_2$ reactions are assumed to be in the range of $k(RO_2 + RO_2) \sim 1 \times 10^{-12} - 10^{-13}$ cm^3 $molecule^{-1}$ s^{-1} and $k(HO_2 + RO_2) \sim 1 \times 10^{-11}$ cm^3 $molecule^{-1}$ s^{-1} [24]. From this, first-order rate coefficients for the stated upper reactant concentration limit are derived as $k^{1st}(RO_2) < 8 \times 10^{-4}$ s^{-1} and $k^{1st}(HO_2) < 8 \times 10^{-3}$ s^{-1}. The corresponding lifetimes are > 21 minutes and > 2.1 minutes, respectively. In the case of NO, a background concentration smaller than 1×10^{8} molecules cm^{-3} can be deduced from the experiments with NO additions. In those, even for an NO addition of 5.6×10^{8} molecules cm^{-3}, clear product signals from $NO + RO_2$ were visible (Supplementary Fig. S9), which were absent without NO additions. An assumed rate coefficient $k(NO + RO_2)$ of 1×10^{-11} cm^3 $molecule^{-1}$ s^{-1} [24] yields a first-order rate coefficients of $< 1 \times 10^{-3}$ s^{-1} for NO concentrations smaller than 1×10^{8} molecules cm^{-3} corresponding to a RO_2-lifetime with respect to the NO reaction of more than 16 minutes.

Consequently, any bimolecular reactions of RO_2 radicals with other RO_2 radicals, HO_2 or NO cannot be competitive with the RO_2 isomerization steps proceeding at a time scale of seconds or less."

At different places in the text it is now stated that bimolecular step were negligible in these sets of experiments.

Reply cont.: **Additional** experiments have been performed in order to probe the closed-shell product formation, i.e. carrying out i) runs with NO addition in the free-jet apparatus and ii) H_2O_2 photolysis experiments with elevated RO_2/HO_2 levels in the TROPOS flow-tube. These experiments are now described in "**Additional experiments with NO additions or with elevated HO_2 and RO_2 concentrations.**"
From the experiments with NO additions it was possible to conclude that the highly oxidized RO_2 formation was not hindered even for NO concentrations of a few 10^{10} molecules cm^{-3}. The results from the experiments with elevated RO_2/HO_2 concentrations were merely used to qualitatively study the corresponding closed-shell product formation. The lack of information about the RO_2 and HO_2 concentration levels limit any further conclusions.
Changes in the text: *The different sets of experiments are now clearly mentioned at different places in the text.*
The explanations regarding the NO additions runs in the text have been broadened. Fig.S9 was revised and now there is also additional information in the figure caption.

"**Additional experiments with NO additions or with elevated HO_2 and RO_2 concentrations.** … The total amount of products indicates that the HO-$C_{10}H_{15}(OO)(OOH)O_2$ radical formation was not disturbed by the NO additions, see Supplementary Fig. S9. Thus, the RO_2 isomerization steps leading to HO-$C_{10}H_{15}(OO)(OOH)O_2$ must be faster than the corresponding RO_2 reactions with NO, which proceed with pseudo first-order rate coefficients of up to 0.28 s^{-1}. This estimate assumes a rate coefficient $k(NO + RO_2)$ of 1×10^{-11} cm^3 $molecule^{-1}$ s^{-1} [24]. Consequently, the rate coefficients of the RO_2 isomerization steps forming the RO_2 radicals **19**, **20** and **29** must be higher than 0.28 s^{-1} in line with the results of theoretical calculations (see Supplementary Information). "

2) RO2 lifetimes in the field. I agree that accurate RO2 lifetimes cannot be deduced from field data - and of course the authors are correct that the atmosphere is not a "well-defined laboratory experiment" - but that does not mean that qualitative conclusions cannot be made! The data clearly show that there is an exceedingly slow decay (on the order of hours) of the RO2 radical in question at night. There are only two ways this can be the case: either there is some sort of nighttime source (somewhere in the local/regional atmosphere), or the radical has a lifetime of several hours. If it's the former, there must be a substantial nighttime OH source, or a pathway to RO2 formation by some other channel; if it's the latter, then these RO2 radicals are so slow to isomerizes that bimolecular reactions with almost certainly win out. Which is it? Regardless, this is highly unusual behavior for OH-derived radicals in the nighttime, and so cannot simply be left unmentioned in the text.

Also, the authors mention in the rebuttal that the bimolecular lifetimes are on the order of minutes. Because of the importance of bimolecular lifetimes to this whole story, this needs to go in the main text as well.

Reply: It is extremely difficult to determine RO_2 lifetimes based on atmospheric trace measurements with time due to the complexity of atmospheric processes. But, a rough estimate of the RO_2 lifetime regarding bimolecular reactions with RO_2, HO_2 and NO yields 1.7 - 170 minutes for the individual steps. Altogether, we have to assume a RO_2 lifetime of a few minutes. Thus, the slow nighttime decay of the HO-$C_{10}H_{15}$(OO)(OOH)O_2 trace with time, consistent with a lifetime in the order of hours, requires another explanation. For that we have to take into account effects, such as air mass changes or mixing layer effects, as well as a small but not negligible OH + terpene reaction with OH produced from ozonolysis. Importantly, the nighttime RO_2 measurement are close to the detection limit of the instrument and the influence of instrumental limitations, e.g. in correctly assigning the signal at m/z 311 to the $C_{10}H_{17}O_7$*NO_3- ion, cannot be fully ruled out. All these effects hamper a conclusive explanation to this effect. However, we do feel that we can rule out the possibility that the slow decay is indicative of extremely long lifetimes of these RO_2.

Changes in the text: *A paragraph is added in the main text stating the bimolecular RO_2 lifetimes.*

Possible explanations for the apparently long RO_2 lifetime of hours are now given.

"Atmospheric lifetimes of RO_2 radicals regarding the reaction with other RO_2, HO_2 and NO are in the range of 1.7 - 170 minutes for the individual reactions assuming trace gas concentrations for non-urban conditions of 10^9 molecules cm^{-3} for RO_2 and HO_2 radicals and NO each, $k(RO_2 + RO_2) \sim 1 \times 10^{-12} - 10^{-13}$, $k(HO_2 + RO_2) \sim 1 \times 10^{-11}$ and $k(NO + RO_2) \sim 1 \times 10^{-11}$ cm^3 $molecule^{-1}$ s^{-1} [24]. Taking all bimolecular steps together, a RO_2 lifetime of a few minutes has to be taken into account. The nocturnal HO-$C_{10}H_{15}$(OO)(OOH)O_2 radical decline as given in Fig. 3a corresponds to a RO_2 lifetime of a few hours. It could be speculated that i) changing air masses with changing trace gas composition or ii) boundary layer effects or iii) the non-zero nighttime OH radical reaction with terpenes influenced the nocturnal RO_2 concentration. Also instrumental uncertainties cannot be totally ruled out for the nighttime measurements close to the detection limit."

Other points

P5, 1st full para: I don't see how autoxidation can explain the McVay et al. (ref. 28) results. Their RO2+HO2 lifetimes were 10s for the high-UV case, 50s for the low-UV case. In the present study the unimolecular lifetime of the RO2 radical is thought to be 10s or longer. It would have to be considerably shorter than that to compete with HO2 reaction, such that yield was independent of UV intensity.
Reply: The formation of the highly oxidized RO_2 radical, HO-$C_{10}H_{15}$(OO)(OOH)O_2, proceeds at a second time scale. Fig. S1 depicts the detected HO-$C_{10}H_{15}$(OO)(OOH)O_2 concentrations as a function of time in the range 3 – 7.9 s. This measurements series clearly shows that HO-$C_{10}H_{15}$(OO)(OOH)O_2 formation takes place within the lowest reaction time of 3 s or less. Theoretical calculations do support this experimental finding. McVay et al. conducted their experiments under conditions of RO_2 + HO_2 lifetimes between 10 and 50 s. Under these conditions the HO-$C_{10}H_{15}$(OO)(OOH)O_2 formation, and consequently the HOM formation generally, was not disturbed because the HO-$C_{10}H_{15}$(OO)(OOH)O_2 generation is clearly faster! So, the HOM formation process for OH + α-pinene, as discovered in our study, can very nicely explain the McVay et al. results, at least qualitatively.

P6, 1st full para: The choice of these two references (32 and 33) to discuss the underprediction of SOA by models seems highly unusual. There is a large body of literature describing such underpredictions, and neither of these articles were the first. The first such article was Heald et al. 2005, GRL 32:L18809 2005. (Also, "Russell" is misspelled.)
Reply: We thank the reviewer for this hint and added the mentioned reference, now ref.32. Also the review article by Hallquist at al. was added to the reference list, now ref. 33. It is to be noted, that reviewer 2 in the previous review round focused on the former refs. 32 and 33, now refs. 34 and 35.
Changes in the text: New references 32 - 35. "Russell" has been corrected.

P7, 2nd para: This discussion is highly repetitive of what is stated previously (top of p. 4). Moreover, it is quite misleading. Refs. 34 and 35 say little to nothing about sequential RO2 isomerizations, but instead focus on the initial isomerization (RO2->QOOH) only. (I don't have access to ref 23.) In combustion, the resulting OOQOOH radical typically doesn't undergo additional isomerization reactions, but rather decomposes, giving OH and other products. (This is actually made very clear in ref 35!) Also, while the combustion community has known about ROO->QOOH reactions for a long time, but vast majority of that work (until recently) was for R=alkyl groups. In that case, the C-H bond is sufficiently strong that isomerization won't happen at 298K – this is why atmospheric chemists, also focused initially on alkane oxidation, did not consider it. Only as the focus has shifted to the oxidation reactions of oxygenates (with weaker C-H bonds) has the importance of such isomerization reactions become clear.
Reply: We still think that the connection to combustion chemistry is worth mentioning. At the same time, we also agree that there is no need to repeat it in the Discussion section.
Changes in the text: The corresponding small paragraph in Discussion was deleted incl. the former refs. 34 and 35. The statement in the main text was slightly reformulated.

“It should be noted that the reaction sequence of RO_2 radical isomerization (H-shift or cyclization) followed by O_2 addition, as outlined in Scheme 1, represents a reaction principle well known in low-temperature combustion chemistry[23].”
